# Metabolic and metagenomic outcomes from early-life pulsed antibiotic treatment

**DOI:** 10.1038/ncomms8486

**Published:** 2015-06-30

**Authors:** Yael R. Nobel, Laura M. Cox, Francis F. Kirigin, Nicholas A. Bokulich, Shingo Yamanishi, Isabel Teitler, Jennifer Chung, Jiho Sohn, Cecily M. Barber, David S. Goldfarb, Kartik Raju, Sahar Abubucker, Yanjiao Zhou, Victoria E. Ruiz, Huilin Li, Makedonka Mitreva, Alexander V. Alekseyenko, George M. Weinstock, Erica Sodergren, Martin J. Blaser

**Affiliations:** 1 Department of Medicine, New York University School of Medicine, New York, New York 10016, USA; 2 Department of Microbiology, New York University School of Medicine, New York, New York 10016, USA; 3 New York Harbor Department of Veterans Affairs Medical Center, New York, New York 10010, USA; 4 The Genome Institute at Washington University, St. Louis, Missouri 63108, USA; 5 Department of Pediatrics, Washington University School of Medicine, St. Louis, Missouri, USA, 63110; 6 Division of Biostatistics, Department of Population Health, NYU Langone Medical Center, New York, New York 10016, USA; 7 Division of Infectious Diseases, Department of Internal Medicine, Washington University School of Medicine, St. Louis, Missouri 63110, USA; 8 Center for Health Informatics and Bioinformatics, New York University School of Medicine, New York, New York 10016, USA; 9 The Jackson Laboratory for Genomic Medicine, Farmington, Connecticut 06117, USA

## Abstract

Mammalian species have co-evolved with intestinal microbial communities that can shape development and adapt to environmental changes, including antibiotic perturbation or nutrient flux. In humans, especially children, microbiota disruption is common, yet the dynamic microbiome recovery from early-life antibiotics is still uncharacterized. Here we use a mouse model mimicking paediatric antibiotic use and find that therapeutic-dose pulsed antibiotic treatment (PAT) with a beta-lactam or macrolide alters both host and microbiota development. Early-life PAT accelerates total mass and bone growth, and causes progressive changes in gut microbiome diversity, population structure and metagenomic content, with microbiome effects dependent on the number of courses and class of antibiotic. Whereas control microbiota rapidly adapts to a change in diet, PAT slows the ecological progression, with delays lasting several months with previous macrolide exposure. This study identifies key markers of disturbance and recovery, which may help provide therapeutic targets for microbiota restoration following antibiotic treatment.

## Abstract (web summary)

The potential recovery of the human gut microbiota after an antibiotic treatment, and its effects on our health, are poorly understood. Here, the authors use a mouse model mimicking paediatric antibiotic use to shed new light into these processes.

## Graphical abstract

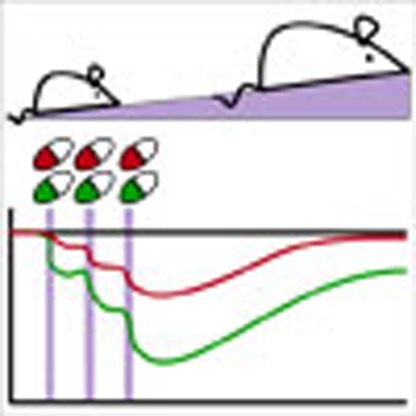

Antibiotic use has reached enormous proportions around the world. In the United States, 262 million courses of antibiotics were prescribed to outpatients in 2011, a rate of 842 courses per 1,000 people annually^1^. Use is highest in children under 10, who receive more than 40 million courses per year^1^,^2^. Extrapolating from prescription data, the average US child receives three antibiotic courses in the first two years of life, and 10 courses by the age of 10 (ref. 1). However, prescription rates are not consistent: within the US, prescription frequency varies by more than 50% across regions^1^, and even more among individual practitioners^3^. In Sweden, antibiotic use is 40% of that in the US^4^. These disparities suggest opportunities to curtail antibiotic use in the US.

A well-known consequence of antibiotic use is the development of bacterial antibiotic resistance^5^. The impact of antibiotics on host and microbial function, on the other hand, has been little studied, as these medications have long been considered extremely safe. The intestinal microbiota aids the host in metabolic and immunological development^6^ and provides beneficial functions such as vitamin production and pathogen displacement. Despite the generally low-risk profile of antibiotics, these medications might be detrimental to host health by disrupting conserved functions of the microbiota, especially when given during critical developmental times. Because the extent of microbiota perturbation can depend on the antibiotic used^7^, characterization of the effects of commonly prescribed antibiotics is needed to develop clinical guidelines.

In addition to directly altering the gut microbiome, antibiotics may impede the ability of the gut microbiota to adapt to stressors. Consumption of a high-fat diet (HFD) is pervasive in westernized societies, including among young children. In murine models, HFD alone alters gut microbiota composition^8^. A second ‘hit’—antibiotic use—might alter the ability of the microbiota to adapt to HFD, modulating the microbial and/or phenotypic effects of HFD.

Since the 1940s, farmers have added low doses of antibiotics to food or water of livestock for growth promotion. The efficacy of this practice has resulted in extensive use^9^. The microbiome is a key mediator of these effects; for example, germ-free chickens do not have the accelerated growth seen in conventionally raised animals exposed to antibiotics^10^, and in other models, manipulating the microbiota through transferring specific organisms can promote weight gain^11^. In prior studies, we examined the effects of such continuous low antibiotic doses (sub-therapeutic antibiotic treatment (STAT)) on the murine intestinal microbiota, showing altered metabolic phenotypes^12^,^13^.

In contrast to farm animals receiving STAT for growth promotion, humans receive 10- to 100-fold higher antibiotic exposures for short courses to treat acute infections^2^. Here, to better understand how human antibiotic use may alter microbial ecology and potentially contribute to the population-wide changes in metabolic development, we develop a mouse model that provides early-life therapeutic-dose pulsed antibiotic treatment (PAT), designed to mimic paediatric antibiotic use. Amoxicillin, a beta-lactam, and tylosin, a veterinary macrolide, were used, as these antibiotic classes are the most commonly prescribed to children^2^. We find that early-life PAT leads to short-term increases in mouse weight and bone growth and longer-term alterations in gut microbiome diversity, composition and metagenomic content that remained months after antibiotic exposure. We provide evidence of the altered metabolic potential by confirming changes to a microbiota-specific metabolic pathway, oxalate degradation^14^, at both metagenomic and metabolite levels. Whether or not antibiotic-mediated effects on growth function through the microbiota, these findings illustrate the potential functional consequences of early-life antibiotic-induced microbial perturbations and highlight the need to re-examine antibiotic guidelines in the human population.

## Results

### Early-life PAT alters murine growth

**Figure 1:**
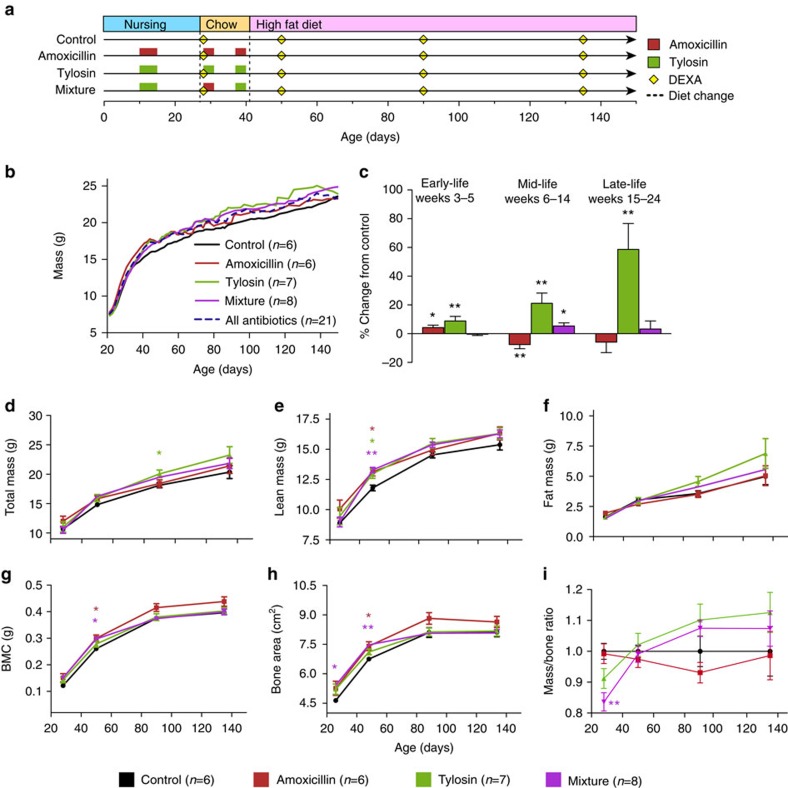
Effect of PAT on growth. (**a**) Timeline of antibiotic pulses and dietary changes. (**b**) Scale weight of mice. (**c**) Growth rates in early-, mid-, and late-life expressed as percent difference from control. (**d–h**) Dual energy X-ray absorptiometry measurements. (**d–f**) Total, lean and fat mass; (**g**) bone mineral content (BMC) and (**h**) bone area. (**i**) Calculated total body mass-to-bone area ratio is represented as a fraction of control. *P<0.05, **P<0.01, ANOVA with Dunnett's post test. (**c–h**) Bars represent standard error of the mean. Number of mice: control, *n*=6; amoxicillin, *n*=6; tylosin, *n*=7 and mixture, *n*=8.

Female C57BL/6J mice received three short courses of therapeutic-dose amoxicillin, tylosin, alternating courses of either antibiotic (mixture) or no antibiotics (control) (Fig. 1a and Supplementary Fig. 1). To mimic early life use by human children, the PAT was completed shortly after weaning, and then mice received high-fat chow to enhance metabolic phenotypes^13^. After one pulse, all mouse groups had identical weights on day 21 (Fig. 1b), but early-life PAT significantly increased the cumulative weight gain from 3 to 6 weeks, an effect continuing through late life in tylosin mice (Fig. 1c and Supplementary Fig. 1b).

Tylosin and amoxicillin PAT had different effects on body composition. Compared with controls, tylosin significantly increased both total and lean mass, while amoxicillin only significantly increased lean mass (Fig. 1d–f). All groups of PAT mice developed larger bones than controls (Fig. 1g,h), but increases in bone area and mineral content were most pronounced in the amoxicillin group, demonstrating body composition variation based on antibiotic class. When mass-to-bone ratio (total body mass/bone area) was calculated as a proxy for body mass index (Fig. 1i), the tylosin and mixture mice both gained mass/area at significantly higher rates than control (*P*=0.015 and 0.005, respectively; longitudinal analysis using the linear spline models). Altogether, these data demonstrate significant early-life PAT-antibiotic-specific growth promotion.

### Effects on host physiology

**Figure 2:**
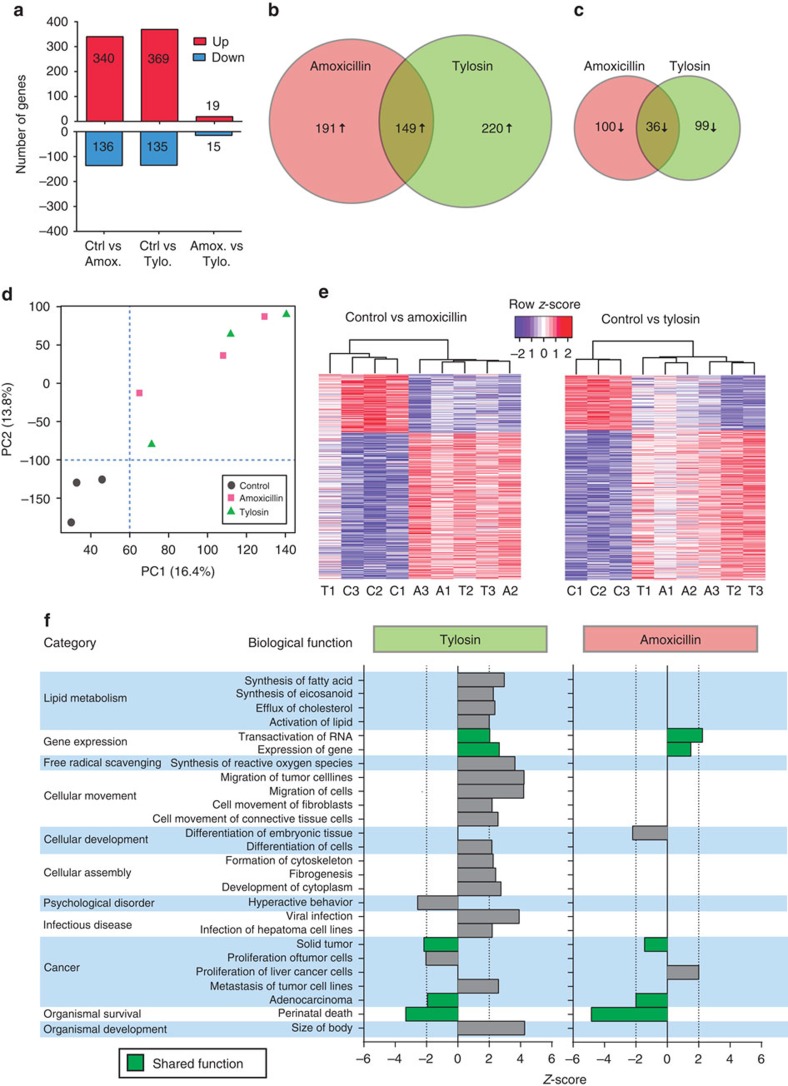
PAT alters hepatic gene expression. (**a**) Number of differentially expressed genes (*P*<0.01 and |log_2_fold change|>0.5) that are up- or down-regulated in the underlined group with respect to the comparator group. (**b**,**c**) Venn diagrams showing numbers of upregulated or downregulated genes, respectively, that are shared or unique. (**d**) Principal component analysis plot of hepatic gene expression data, representing 30.2% of total variation. (**e**) Expression of hepatic genes significantly altered by amoxicillin or tylosin with respect to control. (**f**) Predicted biological functions that are differentially represented (*P*<0.05, z-score |2|) based on Ingenuity Pathway Analysis of hepatic expression. Amox., amoxicillin; Ctrl, control; Tylo., tylosin.

As hepatic metabolism can be altered with early-life antibiotic exposure^13^, potentially as a result of altered growth, direct effect of antibiotics or influence of the microbiota through the enterohepatic circulation, we examined whether we could detect PAT effects on the liver long after antibiotic cessation. Early-life tylosin significantly elevated micro- and overall hepatic steatosis in later life (Supplementary Fig. 2a–e), while amoxicillin significantly reduced microsteatosis. All groups had similar liver mass (Supplementary Fig. 2f). PAT induced changes in hepatic gene expression, with more upregulated than downregulated genes with respect to controls (Fig. 2a). While few genes had been significantly modulated by both the early-life tylosin and amoxicillin exposures (Fig. 2b–d), hierarchical clustering of genes significant in either group (Fig. 2e) indicates similar trends in both antibiotic groups.

Altered biological functions, predicted by Ingenuity Pathway Analysis, revealed antibiotic-specific effects; several pathways, including those related to gene expression, cancer, and organismal survival, were abnormal in both groups (Fig. 2f, green bars). Early-life tylosin exposure also increased expression of genes relating to lipid metabolism and cellular movement and assembly, consistent with the increased steatosis. Both by microarray and qPCR, *Hspb1*, a heat-shock protein reported as microbiota modulated^15^, was significantly downregulated in mice receiving either antibiotic (Supplementary Fig. 2g). The alterations in hepatic gene expression, long after the final antibiotic course, demonstrate that these early-life exposures have metabolic influences, partially conserved across both antibiotic classes, which can be detected long after antibiotic exposure.

To investigate systemic impacts of PAT, metabolic hormones were measured in fasting serum samples at sacrifice. Early-life tylosin or mixture PAT significantly reduced ghrelin compared with controls, whereas amoxicillin showed a lesser effect (Supplementary Fig. 3a–e). Peptide YY, leptin, amylin and insulin did not significantly differ between the groups. As expected, leptin levels were positively correlated with fat mass in control mice alone, PAT mice alone and in all mice (Supplementary Fig. 3g–i). Peptide YY negatively correlated with fat mass in the control mice, as expected, but not in the PAT mice (Supplementary Fig. 3j–l), indicating that PAT had disrupted this metabolic relationship. In all groups, faecal caloric contents decreased over time (Supplementary Fig. 3f), not differing between PAT and control.

### PAT-induced microbiota disruption

**Figure 3:**
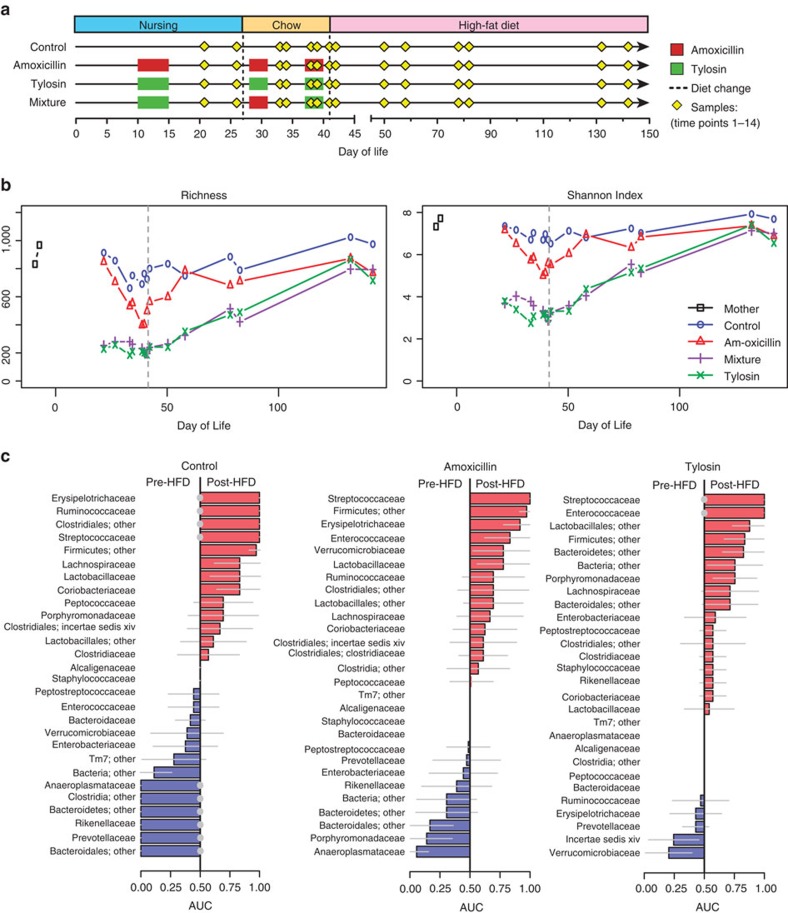
Ecological outcomes from early-life PAT and response to dietary intervention. (**a**) Experimental design and timing of microbiota samples. (**b**) α-diversity measured at a coverage depth of 3,000 sequences per sample. (**c**) Differentiating bacterial families immediately before and after introduction of HFD. Area-under-curve (AUC) with 95% confidence intervals (grey lines) for differentiating pre-HFD and post-HFD mice is plotted by treatment group in major families (>1% relative abundance in at least one mouse). Significantly predictive results (Mann–Whitney test) after false-discovery rate correction (*q*<0.05) are indicated by grey-filled circles.

Microbiota composition was surveyed by sequencing the 16S rRNA gene from serial PAT and control pup faecal pellets and from representative mothers (Fig. 3a), yielding 2,683,548 quality-filtered sequences with a mean±s.d. depth of 6,899±3,009 sequences per sample. The richness and Shannon Index remained relatively constant in control mice, with α-diversity similar to their mothers; however, PAT decreased both richness and Shannon evenness even after one antibiotic pulse, with immediate and sustained reductions in diversity more pronounced following tylosin exposure (Fig. 3b). While amoxicillin resulted in milder reductions, there was a progressive loss with each dose, indicating the importance of both class and number of courses. Although the PAT groups largely converged with control over time, differences never fully resolved.

We next examined compositional changes at the phylum level by qPCR and high-throughput sequencing. Following antibiotic cessation and switch to HFD, Bacteroidetes in the mixture group and some of the tylosin mice were dramatically reduced compared with control, whereas little change was observed in the amoxicillin mice (Supplementary Fig. 4a–d). Beyond changes at the phylum level, the microbiota from mice receiving tylosin or the mixture had significantly different overall microbiota composition from controls (adonis test of unweighted UniFrac distances with Bonferroni-corrected *P*-value<0.05; Supplementary Fig. 4e), detected as early as the first time point (one antibiotic pulse), extending to months after antibiotic cessation. For amoxicillin, a significant shift only was detected after all three pulses, and overall community structure did not differ from controls 1 week after antibiotic cessation and switch to HFD, indicating the much more prolonged effects of the macrolide-based treatments compared with the beta-lactam. Unweighted UniFrac distances by group and cage (Supplementary Fig. 4f) revealed that control microbiota remained relatively homogenous over time (cage 1 and 2); one cage of amoxicillin mice had microbiota similar to control (cage 3), while the other two showed divergence from control either immediately after three pulses of antibiotic (cage 5) or weeks after antibiotic cessation (cage 4). Tylosin and mixture microbiota behaved similarly, regardless of cage, with the greatest divergence after three pulses of antibiotic, and gradually approaching control composition by the end of the experiment. These data indicate that the strong perturbation by macrolide produced consistent effects, whereas the milder disruption by beta-lactam had more variation in microbiota effects.

To identify key members of the microbiota associated with a rapid transition to HFD, we performed area-under-the-curve analysis (Fig. 3c). Especially for the controls, multiple families changed significantly (false-discovery rate-adjusted *P*-value, *q*<0.05, Mann–Whitney *U*-test), either increasing or decreasing after HFD commencement. Members of the phylum Firmicutes have been reported to increase with HFD exposure at the expense of Bacteroidetes^16^, and such patterns were observed in control mice with the increase of Erysipelotricaceae, Ruminococcaceae, Streptococcaceae, unclassified Clostridiales and Firmicutes other, and the decrease of Rikenellaceae, Prevotellaceae, Bacteroidales other and Bacteroidetes other. Many similar families were changed in the amoxicillin mice in the same direction, but were not significant. In tylosin mice, changes were partially in the same direction (Streptococcaceae, Clostridiales other, Firmicutes other and Prevotellaceae) and partially in the opposite direction (Erysipelotrichaceae, Ruminococcaceae, Rikenellaceae, Bacteroidales other and Bacteroidetes other). Thus, the antibiotic exposures modified typical microbiota responses to HFD, with more aberrant responses observed with the macrolide than the beta-lactam.

### PAT delays microbiota maturation

**Figure 4:**
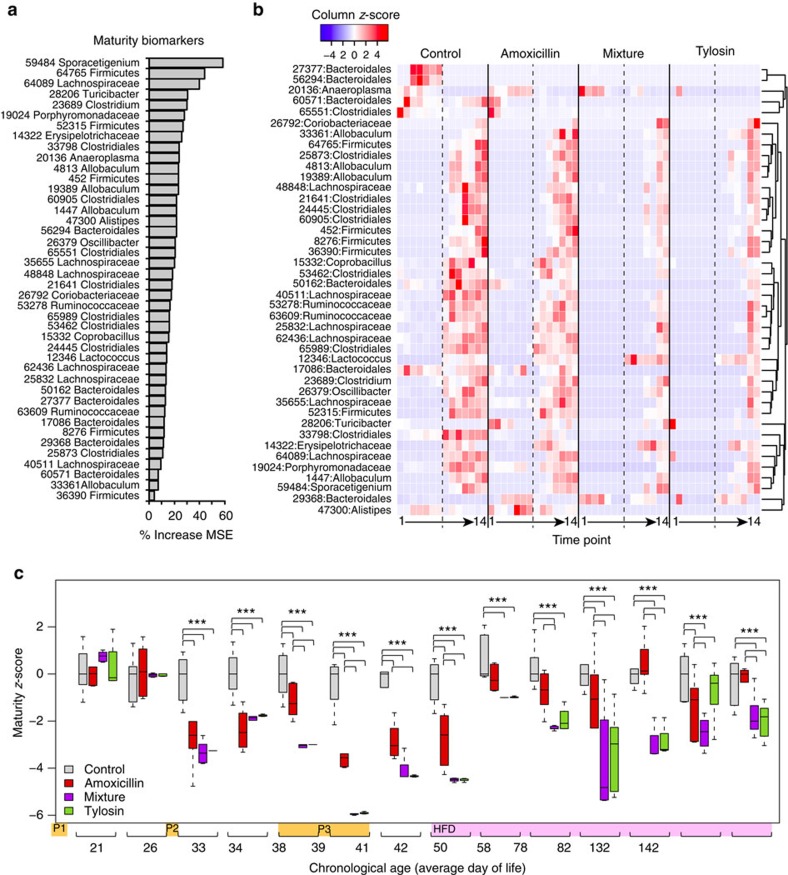
The effect of early-life PAT on microbiota maturity and dietary responses. (**a**) The OTUs that predict maturity and explained the greatest degree of variation in the model, ranked by contribution to reduction of mean square error (MSE). (**b**) Abundance of predictive OTUs over time. Dashed lines indicate introduction of HFD, time points 1–14 correspond to sequential samples (correlating with increasing day of life). (**c**) Average MAZ over time; *z*-score=0 indicates appropriate maturation; higher or lower z-scores indicate accelerated or delayed microbiota development, respectively. *** *P*<0.001 one-way ANOVA with Fisher’s least significant difference adjusted for false-discovery rate.

Because of the importance of the developing microbiota, we compared the relative maturation rates of control and antibiotic-perturbed microbiota using a Random Forests^17^ regression model to predict day of life as a function of microbial composition^18^. Microbial maturity of control samples could be accurately predicted (Supplementary Fig. 5a) using 42 key operational taxonomical units (OTUs; Fig. 4a). Most of these 42 biomarkers showed marked population increases after HFD initiation, with delayed responses in the tylosin and mixture groups (Fig. 4b) accounting for their persistent microbial immaturity through day 142 (Fig. 4c). As the effects of diet and ageing cannot be separated, we constructed a second maturity model for the period of early life before HFD (Supplementary Fig. 5b). In the normally developing (control) mice, several OTUs predominated after weaning, diminished over time and were succeeded by other OTUs. Amoxicillin caused minimal disruption, whereas mixture and tylosin treatment substantially reduced OTUs associated with normal maturation (Supplementary Fig. 5c).

Microbiota-by-age *z*-scores (MAZ)^18^ can quantify delayed or accelerated microbiota development in response to an exposure. Dietary composition strongly influences intestinal microbiota^19^ and thus microbial age predictions; accordingly, some of the control samples immediately shifted after HFD initiation, predicting an older age (Fig. 4c). PAT delayed both maturation and response to HFD, with the greatest effects from tylosin or mixture exposure. The first antibiotic pulse had no effect on microbial maturity, but MAZ dropped substantially after the second pulse, progressively decreasing during the third pulse. The amoxicillin group approached control between pulses, and the MAZ score converged with control after ∼1 week of HFD. The tylosin and mixture groups trended towards control on HFD, but never converged. In total, antibiotic exposure delayed microbiota maturation and response to diet, with both the number of courses and antibiotic class determining the extent of disruption.

### Linking perturbation with outcomes

**Figure 5:**
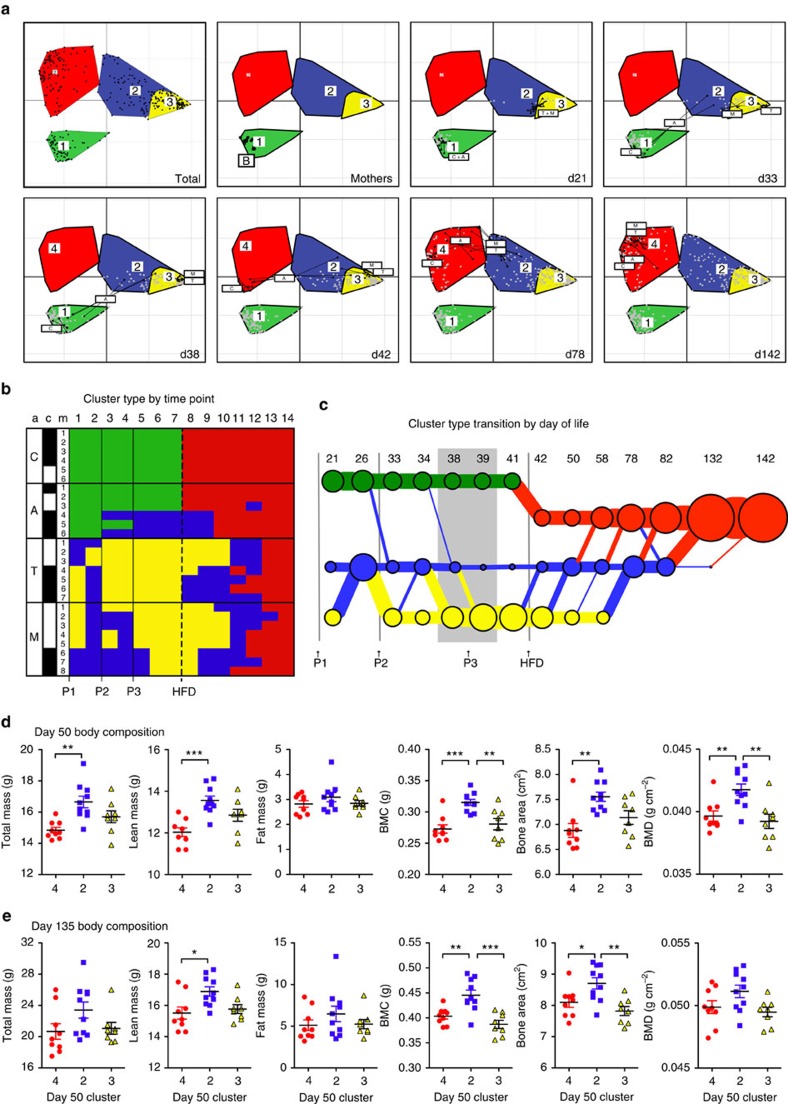
Dynamics of disruption, recovery and response to HFD. (**a**) Community structure over time within the four clusters identified by Calinski analysis shown for mothers and for pups at selected representative time points: after the first, second and third antibiotic pulses and after starting HFD. (**b**) Cluster assignment by mouse and time point. a, antibiotic group; c, cage (bars indicate mice in the same cage); m, mouse. Time points 1–14 correspond to sequential samples (correlating with increasing day of life). (**c**) Microbiota transition map, circles and lines are scaled to represent number of mice in each cluster (circle) or transitioning (line) between clusters. (**d**,**e**), Body composition grouped by day 50 cluster type at ∼50 days of life (**d**) and at ∼135 days of life (**e**). *P<0.05, **P<0.01, ***P<0.001, ANOVA with Tukey-post test. C, control; A, amoxicillin; T, tylosin; M, mother.

Grouping samples by composition facilitates characterization of microbiota perturbations. Following evidence that human microbiota may segregate into clusters^20^, we asked whether the PAT-induced microbial shifts could be similarly characterized, and found that four distinct groups best captured the taxonomic variation (Supplementary Fig. 6a). Offspring control microbiota stably clustered with the maternal samples initially (cluster 1), then shifted to a new community state (cluster 4) immediately following introduction of HFD (Fig. 5a). Amoxicillin samples showed mild disruptions for half of the mice after at least two pulses, but converged with controls in cluster 4 shortly after HFD introduction. In tylosin and mixture mice, one tylosin pulse immediately disrupted microbiota, shifting samples to cluster 2 (mildly altered state) or cluster 3 (markedly altered from control). All samples shifted to cluster 3 following at least two doses of tylosin. As with amoxicillin, the samples eventually converged in cluster 4 following HFD, but this recovery was delayed and followed a distinct trajectory (Fig. 5b,c). These community dynamics indicate that sequential antibiotic treatments were more disruptive than single antibiotic exposure, show that tylosin had greater effect on the gut microbiota than amoxicillin and confirm the strong effect of diet on the microbiota^19^.

Each cluster was characterized by different dominant microbial populations at the phylum and OTU levels (Supplementary Fig. 6b–c). In maternal and normal chow-associated cluster 1, Bacteroidetes (B) and Firmicutes (F) dominated (B>F) and had higher levels of *Lactobacillus* than other clusters. In response to HFD (cluster 4), the B/F proportions reversed, and the genus *Allobaculum*, known to be HFD associated^13^, increased. Similar to HFD, both clusters 2 and 3 (associated with antibiotics) also had F>B, and had large blooms of Verrucomicrobia (*Akkermansia*), small blooms of Proteobacteria (*Enterobacter*) and reductions in other taxa. Bacteroidetes was almost completely lost in the tylosin-associated cluster 3, demonstrating that the composition of this cluster was more deviated from baseline than that of the cluster associated with either amoxicillin or one pulse of tylosin (cluster 2).

Because there was variability in the way mice in each antibiotic group responded to and recovered from antibiotics, we next interrogated whether certain microbiota conformations were associated with changes in body composition. We used the cluster identity at day 50 to categorize how the microbiota recovered from antibiotics and responded to HFD in a short time period, and examined the concurrent and later changes in body composition. We found that mice that had a mild dysbiosis in cluster 2 (including mice receiving amoxicillin, tylosin and mixture) had significantly (*P*<0.05, analysis of variance (ANOVA) with Tukey post-test) higher total and lean mass, and bone mineral content, area and density at day 50 compared with mice that transitioned directly from normal chow cluster 1 to HFD cluster 4 (all control and three amoxicillin mice) (Fig. 5d). Of these changes, elevated lean mass, bone mineral content and bone mineral density remained significantly elevated at day 135 of life (Fig. 5e). Mice in cluster 3 (tylosin and mixture mice), which exhibited more extensive shifts in the microbiota from control, did not have significant changes in body composition from cluster 2, indicating that a larger perturbation may dampen the growth promotion effect.

### PAT alters the intestinal metagenome

In metagenomic analyses of 60 faecal samples subjected to shotgun sequencing at a depth of 5 GB each, all mothers and pre-weaning controls (day 21) segregated together on the basis of hierarchical clustering, with high levels of *Lactobacillus*, while all but one sample from the antibiotic-treated mice segregated separately and had high levels of *A. muciniphila* genes (Supplementary Fig. 7). Most (83.3%) of the 18 samples from tylosin mice aggregated together, with substantial *Lactobacillus* depletion and simultaneous *Enterococcus* expansion. Consistent with the phylogenetic distribution, nearly all tylosin samples aggregated together when clustered by Kyoto Encyclopedia of Genes and Genomes (KEGG) module abundance (Supplementary Fig. 8), with decreases in modules related to glycolysis, gluconeogenesis and tRNA biosynthesis, among others, and increases in modules related to the citric acid cycle and nucleoside (inosine) and amino acid (leucine) biosynthesis, among others. Collectively, these trends provide evidence that PAT, especially tylosin, shaped metabolic gene populations.

**Figure 6:**
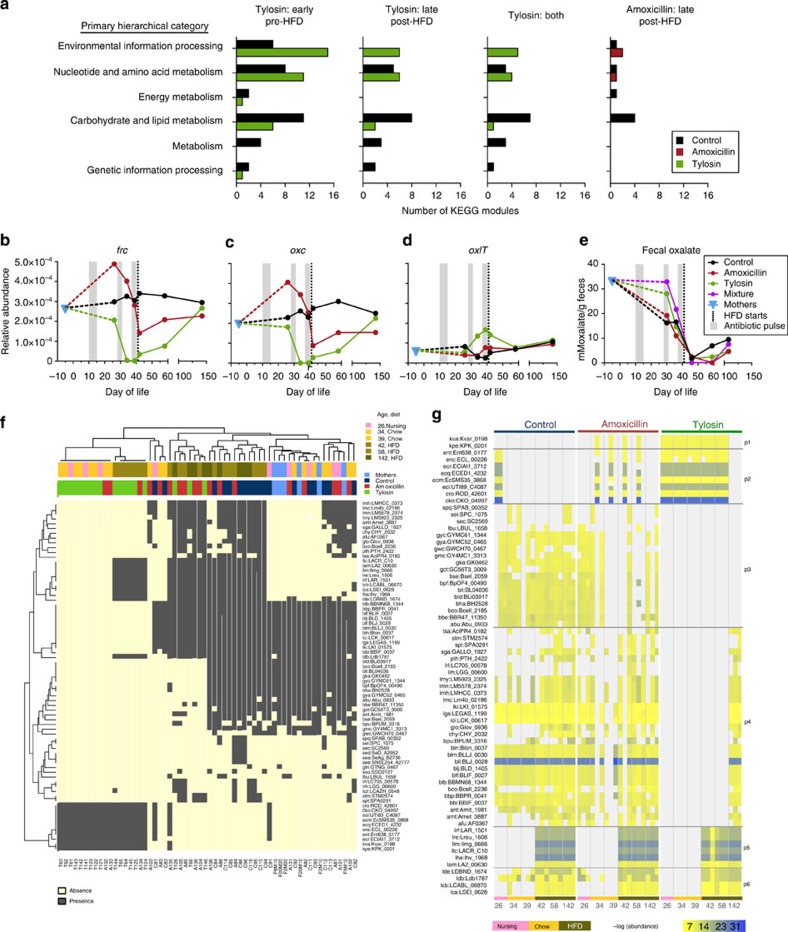
Metagenomic alterations from early-life PAT. (**a**) Number of KEGG modules significantly upregulated in PAT and control mice (*P*<0.05, LEfSe). No modules were significantly different between control and amoxicillin in early life. (**b–d**) Median relative abundance of oxalate metabolism genes over time. (**b**) *frc*, formyl-CoA transferase, (**c**) *oxc*, oxalyl-CoA decarboxylase and (**d**) *oxlT*, oxalate/formate exchanger. (**e**) Faecal oxalate levels. (**f**) Hierarchical clustering of 73 host-associated microbial *oxlT* orthologs (rows) and the associated 60 shotgun sequencing samples (columns). NC, normal chow. (**g**) Relative abundance of 67 *oxlt* orthologs; each cluster group shows a unique presence pattern by condition, diet and time.

To further examine impact on microbiota function, control and PAT metabolic KEGG module abundance was compared using univariate analyses by LEfSe (ref. 21) (Fig. 6a). Compared with control, tylosin significantly affected several metabolic modules in both early-life (normal chow, pre-day 41; Supplementary Fig. 9b) and late-life (HFD, post-day 41; Supplementary Fig. 10b, Supplementary Table 1). Notably, tylosin shifted carbohydrate glycolytic metabolism, depleting the classic Embden–Meyerhoff pathway while increasing the alternate Entner–Doudoroff pathway. Importantly, the early-life tylosin-altered microbial functions related to energy yield from glucose were maintained after antibiotic exposure ceased and diet changed. Amoxicillin also significantly, but less extensively, altered late-life KEGG modules compared with control (Fig. 6a and Supplementary Fig. 10c). Both antibiotics depleted genes related to glycolysis, isoprenoid biosynthesis, tRNA biosynthesis and ribosomes, and enriched genes related to LPS synthesis, proline and vitamin biosynthesis, pyruvate oxidation and molecular transport (Supplementary Table 1). These data indicate metagenomic changes due to the early-life PAT, persisting well into adulthood. The conserved metabolic effects of both antibiotics indicate global, persistent responses to early-life perturbation.

### Microbial oxalate-degrading capacity

To assess the impact of PAT on microbial metabolic capacity, we examined the oxalate-degradation pathway, absent in mammals but well-conserved in bacteria. Oxalate is often present in foods of plant origin, and its metabolism and clearance are critical for urologic health in mammals, which rely on gut microbial species for its digestion. We monitored the three microbial oxalate degradation pathway genes, *frc*, *oxc* and *oxlT* (ref. 22), and found that the relative abundance of the linked genes *oxc* and *frc* showed nearly identical flux over time in all three experimental groups, whereas *oxlT* showed overall lower abundances and unique trajectory (Fig. 6b–d). In the controls, relative ortholog abundances generally remained stable near maternal levels, whereas *frc* and *oxc* varied dramatically in early-life PAT mice. Compared with maternal samples, faecal oxalate levels were reduced during early life in control and amoxicillin pups, but not in the tylosin pups (Fig. 6e), consistent with simultaneous low *oxc* and *frc* relative abundances. Following the second antibiotic pulse, *frc* and *oxc* relative abundances declined further in both antibiotic groups. After HFD initiation, as *frc* and *oxc* abundances normalized, faecal oxalate levels fell in all groups.

Because different orthologous genes within bacterial populations may account for the substantial intergroup *oxlT* relative abundance differences, we examined the responsible variants. Hierarchical clustering (Fig. 6f) showed deep branching, with samples from tylosin mice clustering at one pole and those from mother, pre-weaning control and day 21 amoxicillin mice at the other. The tylosin samples showed unique orthologs not otherwise detectable in control. A large ortholog set, detected uniformly across control and PAT mice, appeared solely after HFD introduction. The relative abundance of the *oxlT* orthologs in the amoxicillin mice over time was similar to controls, whereas tylosin mice were markedly altered in early-life, with partial recovery by late adulthood (Fig. 6g).

Distinct disruption and recovery patterns (p1–p6) could be detected for *oxlT*, which can serve as a proxy for disruption and recovery of broader microbiota functions (Fig. 6g). Pattern p1 contained orthologs exclusive to PAT mice, predominantly in the tylosin mice, disappearing only in the final sample obtained long after antibiotic cessation. In contrast, patterns p3 and p4 included orthologs absent in tylosin mice either entirely or during development, or reestablished at the final time sampled, respectively. Patterns p5 and p6 corresponded to orthologs appearing after HFD introduction in all mice. In total, the substantial flux in early-life (tylosin>amoxicillin) metagenomic content was consistent with the 16S data. Through metagenomic sequencing and metabolite characterization, we detected a microbial pathway strongly influenced by PAT and dietary alterations, differing by antibiotic regimen, with partial functional recovery by gain of redundant genes.

### PAT selects for antimicrobial resistance genes

**Figure 7:**
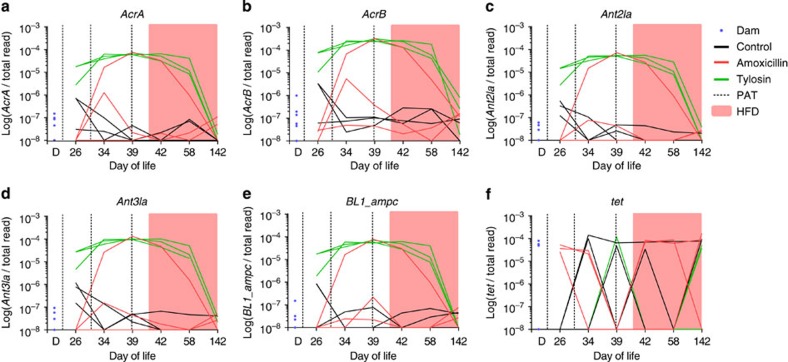
Selection for antibiotic resistance genes in the intestinal metagenome. Frequency of specified antibiotic resistance genes (**a–f**) that were detected in dams, controls, amoxicillin and tylosin mice, shown in relation to the total number of sequence reads for that sample is shown. Dotted lines, antibiotic pulses; pink, HFD.

Multiple classes of antibiotic resistance-associated genes were examined by mapping the metagenomic reads to the resistance gene database. Among genes related to macrolide resistance, four—*acrA*, *acrB*, *ant3Ia* and *ant2Ia—*were present at very low frequency (<10^−7^) among dams (Fig. 7a–d). However, all three mice in the tylosin group showed blooms of these genes to ∼10^−4^, as did one of the mice receiving amoxicillin. No controls showed a change in frequency of these genes. The same mouse receiving amoxicillin and the three tylosin-receiving mice all had blooms of *ampC*, a beta-lactamase gene (Fig. 7e). For 15 tetracycline genes (Fig. 7f) and for hundreds of other genes in the metagenome, there were no differences between mothers, controls and antibiotic-receiving mice, indicating a lack of selection for their resistances.

## Discussion

Antibiotic use is widespread in the US and worldwide, especially in young children^1^, and the development of the early-life microbiota is important for many aspects of health and disease^6^,^18^. In this manuscript, we explore the effects of antibiotics of commonly prescribed classes on gut microbiota maturation, recovery from antibiotics and response to HFD. While we are limited in drawing causal conclusions between microbiota alterations and physiological outcomes, we provide characterization of the microbiota recovery (resilience) after early-life antibiotic exposures.

We created a murine model of early life pulsed antibiotic treatment in conjunction with later HFD to understand the long-term consequences of therapeutic-level antibiotics on microbial ecology, function and ability to adapt to stressors. We found that PAT affected both the host, with early effects on growth and body composition, and the microbiome, with marked metagenomic composition and functional changes, with similar patterns of disruption, recovery and response to HFD in both the 16S and metagenomic data. Consistent with the prior STAT models, in which low-dose (sub-therapeutic) antibiotic treatment was administered over a longer period^12^,^13^, we observed early growth acceleration despite different exposure patterns and antibiotic classes. PAT increased lean mass in all PAT groups, bone in amoxicillin-treated mice, and trended towards increased fat in tylosin-treated mice. Both bone and fat are of mesenchymal origin, suggesting that effects of antibiotic exposure may be partially mediated by altered mesenchymal stem-like cell differentiation^23^. Further studies are needed to explore this hypothesis. Compared with the STAT studies, the gain in fat mass was of lower magnitude and duration, suggesting that therapeutic courses of antibiotics may yield a more limited effect on long-term metabolic outcomes.

Beyond changes in body composition and growth, PAT altered hepatic gene expression and a metabolic hormone level long after antibiotics were stopped, indicating the metabolic effects were systemic. In terms of liver adiposity, tylosin increased microsteatosis, which has been linked with mitochondrial oxidative stress, but not macrosteatosis, which is associated with metabolic aberrations including obesity, insulin resistance, alcoholism and malnutrition^24^. Hepatic changes could be mediated by several factors, including influence from secreted microbial products via the enterohepatic circulation, a direct effect of antibiotics^25^, or an indirect reflection of the altered metabolic status of the rapidly growing PAT mice. These observations raise the possibility that early-life antibiotic treatment may influence metabolic phenotypes in humans if there are similar effects from paediatric exposures.

Unlike observations in agriculture, in which a range of sub-therapeutic antibiotics result in similar growth promotion effects^9^, PAT-mediated effects on host phenotype differed substantially by antibiotic class. Although further studies are needed to explore the mechanism through which antibiotics yield disparate effects, our metabolic studies of oxalate yield initial contributions. Mice receiving amoxicillin had lower faecal oxalate levels than mice receiving tylosin and higher levels of oxalate-degradation genes, indicating removal of a major calcium-binding anion from the gastrointestinal lumen. One explanation for the amoxicillin group having greater bone mineral density than the tylosin group is consistent with enhanced microbial oxalate degradation, which could potentially free calcium for gastrointestinal absorption and augment bone mineral density.

The intestinal microbiota can rapidly shift in response to diet, which may reflect evolutionary advantages for the microbes, host or both. Here the microbiota in control mice rapidly shift to cluster 4 within 1 day of HFD. Conversely, for mice that had already been exposed to a first ecological ‘hit’ of antibiotic treatment, the response to HFD, a second hit, was different: the majority of the PAT mice resisted adapting to the HFD, only shifting to the HFD cluster weeks to months after introduction. Of note, three amoxicillin mice clustered with controls throughout the experiment, demonstrating that when the antibiotic treatment had minimal effects on the microbiota community structure, dietary responses were normal.

Cluster analysis also suggests that the extent of microbiota perturbation may help determine antibiotic-mediated changes in body composition: some perturbation is required for the effect, but too much blunt the effect. Mice whose microbiota were able to rapidly adapt to HFD (cluster 4 one day after HFD initiation), including both control and amoxicillin mice, weighed less than mice with mildly altered microbiota and lagged dietary responses (cluster 2 one week after HFD), suggesting that taxonomic differences could alter physiological outcomes when challenged with HFD. *Allobaculum* that we have previously linked with promoting metabolic health^13^ is depleted, while Lachnospiraceae that we have previously linked with antibiotic-induced obesity^12^ is enriched in cluster 2. That these taxonomic associations are consistent across independent studies with different antibiotic regimens provides support for their potential metabolic involvement. In contrast to mice with mild perturbation, mice with a major disruption (cluster 3 one week after HFD), which included near elimination of the prominent gut phylum Bacteroidetes, did not show elevated weight gain. The intestinal microbiota can contribute to overall energy harvest, and multiple high-dose antibiotics that severely impact the microbiota can lead to weight loss or have no effect on weight^26^,^27^. All three antibiotic regimens were able to lead to mild disruption following HFD, whereas only treatments involving the macrolide led to major disruptions. Thus, we have identified two different potential outcomes from antibiotic treatment with alternate effects on weight. The association between mild disruption and weight gain is consistent with our prior STAT studies, in which low-dose antibiotics lead to a minor microbiota disruption and elevated weight and adiposity^13^.

The dominance of *A. muciniphila* following antibiotic exposure, particularly tylosin, is noteworthy. In humans, *Akkermansia* populations may bloom after antibiotic courses^28^. Levels are inversely correlated with body weight^29^ and bloom in hibernating animals^30^. The ‘probiotic’ administration of *A. muciniphila* has improved glucose tolerance and reduced inflammation in adipose tissues^31^. In contrast, *A. muciniphila* was enriched in the gut microbiota in type 2 diabetes mellitus^32^. The high prevalence of *Akkermansia* in the antibiotic-associated clusters 2 and 3, compared with low levels in the control and HFD-associated clusters 1 and 4, may simply reflect antibiotic perturbation of the microbiome, as in humans^28^. These observations are consistent with our earlier view^33^ that *Akkermansia* may represent an opportunistic microbe that flourishes when ecosystems are disrupted.

Our studies in a mouse model show profound effects of the two most widely prescribed antibiotic classes used in human children^2^ on the microbiota and metagenome, from the earliest dose delivered through the mother’s milk and persisting long after antibiotic cessation. The observed effects have potential clinical implications. For example, both antibiotics selected for microbiota with altered central carbohydrate metabolism characteristics. The decreases in central glycolytic (Embden–Meyerhoff) pathways and aminoacyl-tRNA biosynthesis co-occurred with increases in many nutrient transporters and other carbohydrate-processing pathways, suggesting a global switch in selection from production mode to transport/scavenging mode. Further, PAT, especially tylosin, disrupted oxalate metabolism at both the ortholog and metabolite levels. Changes in intestinal oxalate levels may contribute to a range of clinical syndromes, from impacting serum calcium concentration and bone density (discussed above) to producing hyperoxaluria and an increased risk for kidney stones^14^. Although our functional analysis in this paper focused on oxalate, the fact that changes in gene expression corresponded with changes in measured oxalate excretion provides evidence that changes to the microbiota were not merely substitutional. Future functional studies can expand on this finding to delineate the specific metabolic implications of PAT.

While analyses of mice are not always applicable to humans, the observed phenotypes and reductions in diversity mirror developmental effects of early-life antibiotic use in human cohort studies. For example, adults in developed countries have diminished gut microbiota diversity compared with those from areas without extensive modernization^34^; early-life antibiotic exposures could partially account for those findings^35^,^36^. Four recent epidemiologic studies have shown increased adiposity in children who were exposed to antibiotics early in life^35^,^37^,^38^,^39^. This finding was consistent despite differences in study design, size, geographic location and antibiotics to which children were exposed. Development of animal models to better understand the demonstrated impact of antibiotics on the microbiome and growth and development are therefore critically important.

While there is heightened concern for disrupting the early-life microbiota, antibiotic disruptions in adults may also be important. Adult human studies have similarly demonstrated that different classes of antibiotics impact the gut microbiome to varying degrees (for example, amoxicillin has a less disruptive effect than vancomycin)^7^. Furthermore, antibiotic treatment in adults has been linked to recent weight gain^40^ and increased risk of type 2 diabetes^41^, especially after multiple courses or sustained exposure. Future studies are warranted to examine the impact of PAT at multiple phases of life, early and late.

In addition to effects on metabolism, an altered microbiota could also confer enhanced antibiotic resistance. Our metagenomic studies also provide evidence for the direct and indirect selection of antibiotic resistance genes by both PAT antibiotics, consistent with each antibiotic selecting for microbial strains containing integron(s) expressing multiple resistances. While antimicrobial resistance was not the primary focus of this study, future studies can further elucidate the pattern of gene expansion and recovery over time during and after antibiotic exposure. More information about the ways in which such changes affect the pathogenesis of gastrointestinal infection or host resistance to infection may have clinical implications.

Our study has limitations. While we demonstrate murine phenotypic differences associated with altered taxa and metagenome, causality was not established. This study cannot exclude the possibility that some of the phenotypic effects of PAT resulted directly from antibiotic exposure, rather than from microbiota-mediated changes; for example, macrolides are known to directly impact hepatic physiology^25^. However, the significant overlap in hepatic gene expression profiles in the amoxicillin- and tylosin-exposed mice was not consistent with a macrolide effect alone. In the STAT models, cecal content transplants to germ-free mice transferred growth, adiposity and immunologic phenotypes^13^, establishing the centrality of the microbiota in that antibiotic effects model. Parallel PAT models using germ-free mice will be needed to definitively establish causal relationships. Consideration was given to whether a cage effect contributes to the consistency of microbiota changes among mice exposed to the same antibiotic (Fig. 3d). As expected, co-housed mice were more similar than non-co-housed. However, regardless of cage assignment, all mice that received tylosin (tylosin and mixture groups) were far more similar to each other than to control mice or those that received amoxicillin. That patterns of microbial clustering were consistent based on antibiotic exposure, regardless of caging, providing evidence that cage effect does not explain the observed phenomena.

Pulsed antibiotic treatment affected the metabolism of young mice. Although differences were observed and the overall magnitude was small, both the beta-lactam (amoxicillin) and the macrolide (tylosin) significantly affected early growth, lean muscle mass, bone development and hepatic gene expression. Because the antibiotics used represent the classes most widely prescribed to children, and that our findings were consistent with effects of early life sub-therapeutic antibiotic exposures^12^,^13^, this new model extends hypotheses that early-life antibiotic exposures could have long-term developmental metabolic effects, as supported by animal models^12^,^13^ and human epidemiological studies^26^,^35^,^37^,^38^,^39^. In addition, early-life PAT had extensive effects on the gut microbiota that continued at least 120 days after the last exposure, involving changes in both richness and community structure; an unexpected finding, but clear and consistent. These phenomena require further study, especially to characterize the extent of perturbation and establish whether the microbes or antibiotics are the drivers of physiological changes.

## Methods

### Mouse husbandry

Male and female C57BL/6J mice were obtained at 6 weeks of age from The Jackson Laboratories (Bar Harbor, ME) and bred to produce the study group litters. Mice were weaned at day 27 and separated by sex to retain only females. Runts, defined as mice weighing less than two s.d. below the mean weight of female C57BL/6J mice at day 28 of life (Jackson Laboratory Mouse Phenome Database), were excluded from the experiment. All retained mice within each litter remained in the same treatment group and were co-housed with each other only; mice from separate litters within the same treatment group were maintained in separate cages. Each treatment group included mice from at least two different litters (see Fig. 3d). Mice were maintained on a 12-h light/dark cycle and allowed ad libitum access to food and water. At day 41, mice were switched from standard 10% kcal fat rodent chow (PicoLab Rodent Diet 20; LabDiet, Brentwood, MO) to 45% kcal fat rodent chow (OpenSource Diets D12451; Research Diets, Inc., New Brunswick, NJ) (Supplementary Figure 1). All mouse protocols were approved by the New York University School of Medicine Institutional Animal Care and Use Committee.

### Antibiotic treatment

Mice were divided into four study groups; control mice received no antibiotics, while treatment group mice received three antibiotic courses: at days 10–15, 28–31 and 37–40 of life, amoxicillin, tylosin or mixture (sequential courses of tylosin, amoxicillin and tylosin). Among the macrolides, tylosin was selected owing to its water solubility, stability of solution at room temperature and well-documented pharmacokinetics in animals^42^.

Tylosin tartrate and amoxicillin trihydrate (Sigma Aldrich, St. Louis, MO) were dissolved in distilled deionized water at concentrations of 0.333 and 0.167 mg ml^−1^, respectively, to provide mice with 50 mg of tylosin or 25 mg of amoxicillin per kg body mass per day, based on an estimated daily water consumption of 150 ml per kg body mass. Untreated water, provided by the facility, was acidified to ∼pH 2.7. Experimental antibiotic doses were determined based on pharmacokinetics of typical human paediatric exposures. In human children, amoxicillin dosed at 45 mg kg^−1^ per day achieved a peak serum concentration of 7.4 μg ml^−1^ over an average of 3 days^43^ with an average time to peak of 1–2 h (http://www.accessdata.fda.gov/drugsatfda_docs/label/2008/050542s24,050754s11,050760s10,050761s10lbl.pdf). As the recommended dose for infants ≤3 months of age is 30 mg kg^−1^ per day, very young children are likely exposed to a slightly lower peak serum concentration. In rodents, oral administration of 25 mg kg^−1^ of amoxicillin achieves similar pharmacokinetic parameters, peaking in less than an hour at a concentration of 5.0 μg ml^−1^ in the plasma, with a half life of 0.3 h (refs 44, 45). In human children, the therapeutic macrolide azithromycin dosed at 10 (day 1) and 5 (days 2–5) mg kg^−1^ per day, a recommended regimen for acute otitis media ( http://labeling.pfizer.com/ShowLabeling.aspx?id=511) achieves a peak serum concentration of 0.2 μg ml^−1^ with an average time to peak of 1.8 h (ref. 46). In rodents, oral administration of 50 mg kg^−1^ of tylosin, a veterinary macrolide, achieves similar pharmacokinetic parameters^42^, peaking at 1.5 h at a serum concentration of ≤1.0 and with a half-life of 0.4 h following intravenous injection.

### Body composition and growth rate analyses

Beginning at day 21 of life, body weights were measured daily for the first weeks of life, then weekly in mid- and late-life, on an Ohaus CS200 electronic scale. Body composition, including total body, lean and fat mass, percent body fat, bone mineral content, bone area and bone mineral density, was determined using dual energy X-ray absorptiometry (DEXA) with a Lunar PIXImus II mouse densitometer (GE Medical Systems, Waukesha, WI), as described^12^ on or near days 28, 45, 90 and 135 of life. Anaesthesia was maintained with 0.5% isofluorane in oxygen. Mice were sacrificed by CO_2_ narcosis and cervical dislocation and blood was collected via cardiac puncture and the liver and pancreas were collected. All samples were either fixed in formalin or snap frozen and stored at −80 °C until processing.

### Serum hormone analysis

A protease inhibitor solution contained 0.1 ml protease inhibitor cocktail (AEBSF, aprotinin, bestatin, EDTA, E-64, leupeptin; Sigma Aldrich), 0.1 g 4-(2-aminoethyl) benzenesulfonyl fluoride hydrochloride (AEBSF; Sigma Aldrich), and 0.9 ml dipeptidyl peptidase-4 (DDPIV) inhibitor solution (EMD Millipore Corporation, Billerica, MA). Blood collected at sacrifice was mixed 100:1 with the stock protease inhibitor. Blood cells and serum were separated by centrifugation (1,000*g* for 10 min) and serum was frozen at −70 °C. Serum specimens (100 μl) were examined using a Mouse Gut Hormone Panel (EMD Millipore Corporation) using a Luminex 200 analyser (Millipore). Two-sided Wilcoxon Rank-Sum tests were used for group comparisons.

### Statistical analysis of longitudinal weight and DEXA data

Longitudinal analysis of early-life (weeks 3–5) and mid- and late-life (weeks 6–24) weights were modeled by a linear trend model. For early life weight, the daily group means of mouse weight were fit into the following model:




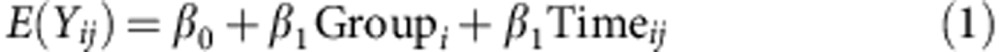




For mid- and late-life weights, the weekly group means of mouse weight were fit into the following linear spline model with common knots at week 15:




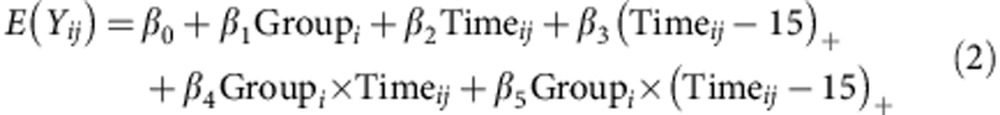




where *Y_ij_* is the weight of *i*th mouse at the *j*th time point; group=0 indicates the control group and Group=1 indicates the PAT group; and (*x*)+ is defined as a function that equals *x* when *x* is positive and is equal to zero otherwise. With the above models, we performed the group comparisons of changing trends over the periods of weeks 3–5, 6–14 and 15–24 (‘early’, ‘mid’ and ‘late’, respectively). Group means of DEXA measurements also were fit to a linear spline model similar to equation (2) with two common knots at week 6 and 15 and analysed over the same ‘early’, ‘mid’ and ‘late’ periods. The MIXED procedures of SAS software (version 9.2; SAS Institute, Inc., Cary, NC) were used to perform the tests and calculate the estimates.

### Statistical analysis of qPCR data

The normality of the data was first tested using the Shapiro–Wilk test. Two sided *t*-test and Wilcoxon Rank-Sum tests were used for group comparison on the normal and non-normal data separately.

### Statistical analysis of serum data

Two-sided Wilcoxon Rank-Sum tests were used for group comparison.

### DNA isolation and analysis

Beginning at day 21 of life, faecal pellets were collected daily for the first weeks of life, and weekly in mid- and late-life, then stored at −20 °C until processing. DNA was extracted from pellets using the PowerSoil DNA isolation kit (Mo Bio Laboratories Inc., Carlsbad, CA) and stored at −20 °C. qPCR was performed in duplicate, using the SYBR Green 1 Master Kit on a LightCycler 480 (Roche Applied Science, Indianapolis, IN). Bacterial standards were cloned into pGEM-T Easy vector following standard procedures and purified using plasmid purification QIAfilter Midi Kit (QIAGEN Inc., Valencia, CA), using described protocols and primers for total bacteria, Bacteroidetes and Firmicutes^47^. For fixed time point statistical comparisons, a Shapiro–Wilk normality test was first performed on each qPCR variable for each group. Two-sided *t*-tests and Wilcoxon Rank-Sum tests were used for group comparisons on the normal and non-normal data, respectively.

### Histology and immunohistochemistry

Liver and pancreas tissue specimens were collected into formalin at sacrifice. Specimens were processed on a Tissue Tek VIP E150/300 tissue processor (Sakura Finetek USA Inc., Torrance, CA) and embedded in paraffin. Digital image analysis of liver samples was performed as described^48^. Hematoxylin–and-eosin-stained liver sections were assessed blindly and independently by two readers. Ten arbitrarily selected images at × 200 magnification for each mouse liver biopsy were produced to ensure a representative sample for each specimen. Extent of steatosis, lobular inflammation and hepatocyte ballooning were assessed using the non-alcoholic fatty liver disease Activity Scoring System^49^. Hepatocyte percent in each specimen exhibiting any degree of steatosis was assigned a score as follows: 0=<5%, 1=5–14%, 2=15–29%, 3=30–65% and 4=>65%, as described. Micro- and macro-vesicular steatosis were defined as diameter ≤15 and >15 μm, respectively. Steatosis with diameter ≤1 μm, difficult to differentiate from staining artifacts or heptocyte ballooning, was excluded from the scoring assessment. Steatosis, lobular inflammation and hepatocyte ballooning also were scored using the non-alcoholic fatty liver disease Activity Scoring System by a third reader examining the entirety of each liver specimen at × 200 magnification. Hematoxylin-and-eosin-stained pancreas sections were similarly assessed blindly and independently by two readers, counting the number of islets per slide and measuring the diameter of the largest islet in each slide and the number of constituent cells.

### Liver gene expression

Liver sections collected at sacrifice were kept overnight in RNAlater at 4 °C and then stored dry at −80 °C. Total RNA was extracted using the RNeasy Mini Kit (Qiagen, Germantown, MD), according to the manufacturer’s instructions. Total RNA quantity was determined by Nanodrop ND-1000 and quality was determined by agarose gel and the Agilent 2100 bioanalyzer. Total RNA was used to prepare cDNA following the 3′ IVT Express Kit labelling protocol (Affymetrix, Santa Clara, CA) and hybridized to the Affymetrix Mouse Genome 430 2.0 Array chip (Affymetrix, Santa Clara, CA) for expression profiling of PAT and control groups. Microarray data were analysed using the Limma package in the R interface. The raw microarray data were normalized using the robust multi-array average method^50^. Differentially expressed genes were found using the empirical Bayes moderated *t*-statistics^51^. Probes with *P*-value<0.01 and |log_2_fold change| >0.5 were considered to be differentially expressed. Alterations in predicted biological functions were detected with Ingenuity Pathway Analysis (Qiagen, Germantown, MD).

For validation by qPCR, 1 μg of total RNA was reverse transcribed to cDNA using SuperScript II reverse transcriptase (Applied Biosystems). To design primers for genes of interest and housekeeping genes, Ensembl and Primer3 were used. Primer pairs were designed to span the intron closest to the 3′ end of the simplest protein-coding transcript. qPCR was performed on a 384-well plate with Power SYBR Green (Applied Biosystems) and run on a ViiA7 Real Time qPCR system (Applied Biosystems), using 0.5 μM primer concentration and 75 ng of cDNA. Target mRNA was normalized using the ΔΔ*C*t method. All target genes were normalized to GAPDH mRNA. Liver Glyceraldehyde 3-phosphate dehydrogenase (GAPDH) and Hspb1 cDNA transcripts were quantified by RT–qPCR using the Roche LightCycler480 with the following cycling parameters: 95 °C 5 min, then 45 cycles of 95 °C 10 s, 60 °C 10 s and 72 °C 20 s. Primers for GAPDH were F: 3′-TGGTGAAGGTCGGTGTGAAC-5′, R: 3′-CCATGTAGTTGAGGTCAATGAAGG-5′, and primers for Hspb1 were F: 3′-GGCTACATCTCTCGGTGCTT-5′, R: 3′-CTCAGGGGATAGGGAAGAGG-5′.

### Bomb calorimetry

Faecal calories were measured using a Parr 6725 Semi-micro calorimeter. Faecal pellets obtained during experiment days 31, 69, 101, 123 and 160 were dehydrated overnight at 56 °C with a silica gel desiccant. The calorimeter was calibrated at the start of each day’s run using a benzoic acid standard to determine the energy equivalent value. After the experimental calorimetric measurements were obtained, the energy equivalent value was verified using a second benzoic acid standard to ensure intra-run machine consistency. The final results were corrected for fuse length and expressed as calories per gram of dry sample weight.

### Faecal oxalate assay

Faecal oxalate levels were measured by enzymatic determination (Trinity Biotech Oxalate Kit, Jamestown, NY). Individual faecal pellets were lyophilized for 24 h and then rehydrated with 500 μl of water, vortex-mixed, acidified to pH<1 with 10N HCl, vortex-mixed, centrifuged at 10,000*g* for 15 min, and the supernatant was used for oxalate detection. All reagent and sample amounts were adjusted to 0.1 × of the kit protocol. Each sample was run in duplicate, absorbance was measured at 595 nm, and the means for duplicate samples normalized to dry weight.

### Metagenomics

Faecal pellets from three representative mice from the control, tylosin and amoxicillin groups at six time points each, as well as six pre-birth samples from mothers, underwent metagenomic analysis generating 5 GB/sample for a total of 300 GB. Shotgun samples were barcoded and introduced to the Illumina HiSeq instrument to produce 100 base-paired end reads (Supplementary Table 2). Sequences for each sample were filtered for contaminants from the mouse genome using bwa default parameters and for low-complexity regions using dust^52^. Reads were aligned^53^ to a reference genome database including bacterial, archaeal, lower eukaryotic and viral genomes, and a phylogenetic map was developed. In brief, the cleaned reads were aligned to a database of nearly 6,000 reference genome sequences as described^54^ using CLC (CLCbio) with the parameters –l 0.75 –s 0.8, requiring alignments to meet an 80% id+75% aligned length cutoff. The breadth (percent of covered bases over the length of the reference genome) and depth (sum of the depths of each covered base divided by the length of the genome) of coverage were calculated based on all alignments of each genome represented in the database using RefCov ( http://gmt.genome.wustl.edu/packages/refcov/index.html). The genomes with substantial alignments (>1% depth and 1% breadth) were accepted. The coverage values were then normalized to aligned reads to calculate depth of coverage per million reads values. The depth of coverage per million reads values were clustered using the Manhattan distance metric with R and the resulting newick tree was visualized in iTOL^55^.

Metagenomic shotgun reads from each sample were searched against the KEGG gene database (version 58) using Mblastx^56^ with the following parameters "-M 30 -m 20 -e 1 -f S ". The search results were run through HUMAnN^57^, a pipeline developed for obtaining enzyme and pathway abundance and coverage from metagenomic communities. Differentially expressed enzymes and pathways were identified using LEfSe^21^ with default parameters.

### Metagenomic analysis of microbial oxalate metabolism

To perform functional analysis of microbial oxalate degradation, query contigs were assembled using the CLC de novo assembler as noted above, using all reads mapping to the three oxalate metabolizing genes (*frc*, *oxc* and *oxlT*) present in the KEGG orthology compendium. The resulting contigs were mapped using BLASTX^58^ against all microbial sequences stored in KEGG (as of December 2012) that satisfied quality fitness scores. Output was generated in BLASTX format 8 (‘*-mformat 8*’ BLASTX parameter), as FASTA data files collected on a per-sample basis. Output files were processed via HUMAnN^57^ to obtain relative abundance of the corresponding KEGG orthologous oxalate metabolizing genes (referred as genomic identifiers (GIDs)). To determine most impactful (dietary or antibiotic) environmental factors, hierarchical cluster analysis of ortholog presence was performed based on Euclidean distance of GID using the heatmap function, part of the R core package^59^. To visualize relative abundance of presence patterns, abundance quantification as output by HUMAnN was formatted and generated using the R function ggplot2 (ref. 60). Quantitation of total GID fluctuation of the oxalate-metabolizing genes by experimental group and by time point was performed using the Python scripting language, processing raw output from HUMAnN into R-compatible data tables.*****

### Microbial 16S informatics

For each of the study mice, 14 timed samples were studied and several samples from the mothers also were examined, for a total of 338 samples. The methods used for 16S rRNA sequencing were those of the Human Microbiome Project^61^. Roche 454 sequencing of the V3–V5 regions on the 454 FLX platform was performed. The average number of reads was nearly 7,000 per sample, which was sub-sampled to an average of 3,000 reads to reduce the variation in sampling per sample. The downstream processing of 16S rRNA sequences was performed as previously described^62^. In short, the quality-filtered sequences were clustered in QIIME v1.3.0^63^ into 97% identity OTUs. The clusters and representative sequences were determined using UCLUST programme^64^, followed by taxonomy assignment using the RDP Classifier^65^ executed at 80% bootstrap confidence cutoff. Phylogenetic relationship between the OTUs was determined by application of FASTTREE to the PyNAST^66^ alignment of the representative sequences with the Greengenes core-set alignment template. The obtained phylogenetic tree and abundance tables were used to calculate unweighted and weighted UniFrac β diversity indices^67^. The OTU absolute abundance table and UniFrac β diversity matrices were extracted from the pipeline for further analysis in the R statistical programming environment^59^.

### Microbiota clustering

We followed the approach of Arumugam *et al.*^20^ to cluster the microbial communities using the partitioning around medoids method^68^ on the square root of the Jensen–Shannon divergence distances. The Calinski–Harabasz index^69^ was used to establish the optimal number of clusters. The clustering was visualized on the principal coordinate analyses using R package ade4 (ref. 70).

### Microbiota maturity analysis

A random forests^17^ regression model was trained on the control microbiota over the course of the experiment to predict chronological age as a function of microbial composition, as described^18^. Each model was built growing 10,000 trees per forest and *n*/3 variables (OTUs) randomly sampled at each split, where *n* is the total number of OTUs in each model. The model was first generated using all OTUs, then refined using 100-fold cross-validation to determine the minimum number of predictive OTUs required to minimize model error, based on % decrease in mean square error. From this, 42 OTUs were selected to train the final model and explained 81.3% of the total variation of the model. OTU importance was ranked by the % increase in mean square error that occurs when that OTU is removed from the model. The maturity index model was used to predict day of life based on microbiota composition. The mouse age predicted by the model (microbiota age) was used to calculated microbial maturity and MAZ as described^18^, using the following formulae:

Microbial maturity (MM)=microbiota age−median microbiota age of control mice of similar age.

MAZ=MM/s.d. of microbiota age of control mice of similar age.

Significant differences in average MAZ for control and PAT mice at each time point were calculated with one-way ANOVA, followed by Fisher’s least significant difference tests with false-discovery rate error correction. OTU relative abundances were plotted on a heatmap using R package heatmap.2.

### Antimicrobial resistance gene analysis

Host contamination free and high-quality metagenomic shotgun reads were aligned to the Antibiotic Resistance Genes Database ( http://ardb.cbcb.umd.edu/) using RAPSearch2 ( http://omics.informatics.indiana.edu/mg/RAPSearch2/), a translated alignment tool. Resistance gene reads were defined if reads were at least 90% identity to the reference at amino acid level and 75% of the read length was mapped to the reference.

## Additional information

**Accession codes:** The microbial 16S rRNA and metagenomic data have been deposited in the SRA database under the project number PRJNA283552. The liver microarray data have been deposited in the GEO database under accession code GSE68603.

**How to cite this article:** Nobel, Y. R. *et al.* Metabolic and metagenomic outcomes from early-life pulsed antibiotic treatment. *Nat. Commun.* 6:7486 doi: 10.1038/ncomms8486 (2015).

## Supplementary Material

Supplementary Information Supplementary Figures 1-10 and Supplementary Tables 1-2

## References

[b1] Hicks L. A. *et al.* US outpatient antibiotic prescribing variation according to geography, patient population, and provider specialty in 2011. Clin. Infect. Dis. 60, 1308–1316 (2015). 25747410 10.1093/cid/civ076

[b2] Hersh A. L., Shapiro D. J., Pavia A. T. & Shah S. S. Antibiotic prescribing in ambulatory pediatrics in the United States. Pediatrics 128, 1053–1061 (2011). 22065263 10.1542/peds.2011-1337

[b3] Gerber J. S. *et al.* Variation in antibiotic prescribing across a pediatric primary care network. J. Pediatr. Infect. Dis. Soc. piu086 (2014). 10.1093/jpids/piu086 PMC6281136 26582868

[b4] Ternhag A. & Hellman J. More on U.S. outpatient antibiotic prescribing, 2010. N. Engl. J. Med. 369, 1175–1176 (2013). 10.1056/NEJMc1306863 24047078

[b5] Dellit T. H. *et al.* Infectious Diseases Society of America and the Society for Healthcare Epidemiology of America guidelines for developing an institutional program to enhance antimicrobial stewardship. Clin. Infect. Dis. 44, 159–177 (2007). 17173212 10.1086/510393

[b6] Rautava S., Luoto R., Salminen S. & Isolauri E. Microbial contact during pregnancy, intestinal colonization and human disease. Nat. Rev. Gastroenterol. Hepatol. 9, 565–576 (2012). 22890113 10.1038/nrgastro.2012.144

[b7] Vrieze A. *et al.* Impact of oral vancomycin on gut microbiota, bile acid metabolism, and insulin sensitivity. J. Hepatol. 60, 1–8 (2014). 24316517 10.1016/j.jhep.2013.11.034

[b8] Carmody R. N. *et al.* Diet dominates host genotype in shaping the murine gut microbiota. Cell Host Microbe 17, 72–84 (2015). 25532804 10.1016/j.chom.2014.11.010 PMC4297240

[b9] Dibner J. J. & Richards J. D. Antibiotic growth promoters in agriculture: history and mode of action. Poult. Sci. 84, 634–643 (2005). 15844822 10.1093/ps/84.4.634

[b10] Coates M. E., Fuller R., Harrison G. F., Lev M. & Suffolk S. F. A comparison of the growth of chicks in the Gustafsson germ-free apparatus and in a conventional environment, with and without dietary supplements of penicillin. Br. J. Nutr. 17, 141–150 (1963). 14021819 10.1079/bjn19630015

[b11] Nurmi E. & Rantala M. New aspects of salmonella infection in broiler production. Nature 241, 210–211 (1973). 4700893 10.1038/241210a0

[b12] Cho I. *et al.* Antibiotics in early life alter the murine colonic microbiome and adiposity. Nature 488, 621–626 (2012). 22914093 10.1038/nature11400 PMC3553221

[b13] Cox L. M. *et al.* Altering the intestinal microbiota during a critical developmental window has lasting metabolic consequences. Cell 158, 705–721 (2014). 25126780 10.1016/j.cell.2014.05.052 PMC4134513

[b14] Goldfarb D. S. Microorganisms and calcium oxalate stone disease. Nephron Physiol. 98, p48–p54 (2004). 15499215 10.1159/000080264

[b15] Kojima K. *et al.* Enteric flora and lymphocyte-derived cytokines determine expression of heat shock proteins in mouse colonic epithelial cells. Gastroenterology 124, 1395–1407 (2003). 12730879 10.1016/s0016-5085(03)00215-4

[b16] Turnbaugh P. J. *et al.* An obesity-associated gut microbiome with increased capacity for energy harvest. Nature 444, 1027–1131 (2006). 17183312 10.1038/nature05414

[b17] Breiman L. Random forests. Mach. Learn. 45, 5–32 (2001).

[b18] Subramanian S. *et al.* Persistent gut microbiota immaturity in malnourished Bangladeshi children. Nature 510, 417–421 (2014). 24896187 10.1038/nature13421 PMC4189846

[b19] David L. A. *et al.* Diet rapidly and reproducibly alters the human gut microbiome. Nature 505, 559–563 (2014). 24336217 10.1038/nature12820 PMC3957428

[b20] Arumugam M. *et al.* Enterotypes of the human gut microbiome. Nature 473, 174–180 (2011). 21508958 10.1038/nature09944 PMC3728647

[b21] Segata N. *et al.* Metagenomic biomarker discovery and explanation. Genome. Biol. 12, R60 (2011). 21702898 10.1186/gb-2011-12-6-r60 PMC3218848

[b22] Abratt V. R. & Reid S. J. Oxalate-degrading bacteria of the human gut as probiotics in the management of kidney stone disease. Adv. Appl. Microbiol. 72, 63–87 (2010). 20602988 10.1016/S0065-2164(10)72003-7

[b23] Reynolds L. P. & Caton J. S. Role of the pre- and post-natal environment in developmental programming of health and productivity. Mol. Cell. Endocrinol. 354, 54–59 (2012). 22154989 10.1016/j.mce.2011.11.013 PMC3306485

[b24] Tiniakos D. G., Vos M. B. & Brunt E. M. Nonalcoholic fatty liver disease: pathology and pathogenesis. Annu. Rev. Pathol. 5, 145–171 (2010). 20078219 10.1146/annurev-pathol-121808-102132

[b25] Kanoh S. & Rubin B. K. Mechanisms of action and clinical application of macrolides as immunomodulatory medications. Clin. Microbiol. Rev. 23, 590–615 (2010). 20610825 10.1128/CMR.00078-09 PMC2901655

[b26] Cox L. M. & Blaser M. J. Antibiotics in early life and obesity. Nat. Rev. Endocrinol. 11, 182–190 (2015). 25488483 10.1038/nrendo.2014.210 PMC4487629

[b27] Dubos R., Schaedler R. W. & Costello R. L. The effect of antibacterial drugs on the weight of mice. J. Exp. Med. 117, 245–257 (1963). 19867224 10.1084/jem.117.2.245 PMC2137609

[b28] Dubourg G. *et al.* High-level colonisation of the human gut by Verrucomicrobia following broad-spectrum antibiotic treatment. Int. J. Antimicrob. Agents 41, 149–155 (2013). 23294932 10.1016/j.ijantimicag.2012.10.012

[b29] Everard A. *et al.* Crosstalk between Akkermansia muciniphila and intestinal epithelium controls diet-induced obesity. Proc. Natl Acad. Sci. USA 110, 9066–9071 (2013). 23671105 10.1073/pnas.1219451110 PMC3670398

[b30] Carey H. V., Walters W. A. & Knight R. Seasonal restructuring of the ground squirrel gut microbiota over the annual hibernation cycle. Am. J. Physiol. Regul. Integr. Comp. Physiol. 304, R33–R42 (2013). 23152108 10.1152/ajpregu.00387.2012 PMC3543654

[b31] Shin N.-R. *et al.* An increase in the Akkermansia spp. population induced by metformin treatment improves glucose homeostasis in diet-induced obese mice. Gut 63, 727–735 (2013). 23804561 10.1136/gutjnl-2012-303839

[b32] Qin J. *et al.* A metagenome-wide association study of gut microbiota in type 2 diabetes. Nature 490, 55–60 (2012). 23023125 10.1038/nature11450

[b33] Cox L. M. & Blaser M. J. Pathways in microbe-induced obesity. Cell Metab. 17, 883–894 (2013). 23747247 10.1016/j.cmet.2013.05.004 PMC3727904

[b34] Yatsunenko T. *et al.* Human gut microbiome viewed across age and geography. Nature 486, 222–227 (2012). 22699611 10.1038/nature11053 PMC3376388

[b35] Bailey L. C. *et al.* Association of antibiotics in infancy with early childhood obesity. JAMA Pediatr. 168, 1063–1069 (2014). 25265089 10.1001/jamapediatrics.2014.1539

[b36] Blaser M. J. Who are we? Indigenous microbes and the ecology of human diseases. EMBO Rep. 7, 956–960 (2006). 17016449 10.1038/sj.embor.7400812 PMC1618379

[b37] Ajslev T. A., Andersen C. S., Gamborg M., Sørensen T. I. A. & Jess T. Childhood overweight after establishment of the gut microbiota: the role of delivery mode, pre-pregnancy weight and early administration of antibiotics. Int. J. Obes. (Lond.) 35, 522–529 (2011). 21386800 10.1038/ijo.2011.27

[b38] Azad M. B., Bridgman S. L., Becker A. B. & Kozyrskyj A. L. Infant antibiotic exposure and the development of childhood overweight and central adiposity. Int. J. Obes. (Lond.) 38, 1290–1298 (2014). 25012772 10.1038/ijo.2014.119

[b39] Trasande L. *et al.* Infant antibiotic exposures and early-life body mass. Int. J. Obes. (Lond.) 37, 16–23 (2013). 22907693 10.1038/ijo.2012.132 PMC3798029

[b40] Thuny F. *et al.* Vancomycin treatment of infective endocarditis is linked with recently acquired obesity. PLoS ONE 5, e9074 (2010). 20161775 10.1371/journal.pone.0009074 PMC2818846

[b41] Boursi B., Mamtani R., Haynes K. & Yang Y. X. The effect of past antibiotic exposure on diabetes risk. Eur. J. Endocrinol. 172, 639–648 (2015). 25805893 10.1530/EJE-14-1163 PMC4525475

[b42] Lewicki J. Tylosin A review of pharmacokinetics, residues in food animals and analytical methods. *United Nations Food and Agriculture Organization* ftp://ftp.fao.org/ag/agn/food/tylosin_2006.pdf (2006).

[b43] Fonseca W., Hoppu K., Rey L. C., Amaral J. & Qazi S. Comparing pharmacokinetics of amoxicillin given twice or three times per day to children older than 3 months with pneumonia. Antimicrob. Agents Chemother. 47, 997–1001 (2003). 12604533 10.1128/AAC.47.3.997-1001.2003 PMC149282

[b44] Andes D. & Craig W. A. In vivo activities of amoxicillin and amoxicillin-clavulanate against streptococcus pneumoniae: application to breakpoint determinations. Antimicrob. Agents Chemother. 42, 2375–2379 (1998). 9736566 10.1128/aac.42.9.2375 PMC105836

[b45] Du X., Li C., Sun H. K., Nightingale C. H. & Nicolau D. P. A sensitive assay of amoxicillin in mouse serum and broncho-alveolar lavage fluid by liquid–liquid extraction and reversed-phase HPLC. J. Pharm. Biomed. Anal. 39, 648–652 (2005). 15935600 10.1016/j.jpba.2005.04.021

[b46] Nahata M. C., Koranyi K. I., Luke D. R. & Foulds G. Pharmacokinetics of azithromycin in pediatric patients with acute otitis media. Antimicrob. Agents Chemother. 39, 1875–1877 (1995). 7486938 10.1128/aac.39.8.1875 PMC162845

[b47] Guo X., Xia X., Tang R. & Wang K. Real-time PCR quantification of the predominant bacterial divisions in the distal gut of Meishan and Landrace pigs. Anaerobe 14, 224–228 (2008). 18524640 10.1016/j.anaerobe.2008.04.001

[b48] Levene A. P. *et al.* Quantifying hepatic steatosis–more than meets the eye. Histopathology 60, 971–981 (2012). 22372668 10.1111/j.1365-2559.2012.04193.x

[b49] Kleiner D. E. *et al.* Design and validation of a histological scoring system for nonalcoholic fatty liver disease. Hepatology 41, 1313–1321 (2005). 15915461 10.1002/hep.20701

[b50] Irizarry R. A. Summaries of Affymetrix GeneChip probe level data. Nucleic Acids Res. 31, 15e (2003). 10.1093/nar/gng015 PMC150247 12582260

[b51] Smyth G. K. Linear models and empirical bayes methods for assessing differential expression in microarray experiments. Stat. Appl. Genet. Mol. Biol. 3, 1–25 (2004). 10.2202/1544-6115.1027 16646809

[b52] Hancock J. M. & Armstrong J. S. SIMPLE34: an improved and enhanced implementation for VAX and Sun computers of the SIMPLE algorithm for analysis of clustered repetitive motifs in nucleotide sequences. Comput. Appl. Biosci. 10, 67–70 (1994). 7514951 10.1093/bioinformatics/10.1.67

[b53] Li H. & Durbin R. Fast and accurate short read alignment with Burrows–Wheeler transform. Bioinformatics 25, 1754–1760 (2009). 19451168 10.1093/bioinformatics/btp324 PMC2705234

[b54] Martin J. *et al.* Optimizing read mapping to reference genomes to determine composition and species prevalence in microbial communities. PLoS ONE 7, e36427 (2012). 22719831 10.1371/journal.pone.0036427 PMC3374613

[b55] Letunic I. & Bork P. Interactive Tree Of Life v2: online annotation and display of phylogenetic trees made easy. Nucleic Acids Res. 39, W475–W478 (2011). 21470960 10.1093/nar/gkr201 PMC3125724

[b56] Davis C. mBLAST: keeping up with the sequencing explosion for (meta) genome analysis. J. Data Mining Genomics Proteomics 4, 135 (2013). 26500804 10.4172/2153-0602.1000135 PMC4612494

[b57] Abubucker S. *et al.* Metabolic reconstruction for metagenomic data and its application to the human microbiome. PLoS Comput. Biol. 8, e1002358 (2012). 22719234 10.1371/journal.pcbi.1002358 PMC3374609

[b58] Zhao K. & Chu X. G-BLASTN: accelerating nucleotide alignment by graphics processors. Bioinformatics 30, 1384–1391 (2014). 24463183 10.1093/bioinformatics/btu047

[b59] R_Development_Core_Team. R: A language and environment for statistical computing R Foundation for Statistical Computing (2012).

[b60] Wickham H. ggplot2: elegant graphics for data analysis Springer (2009).

[b61] Human Microbe Project Consortium. Framework for human microbiome research. Nature 486, 215–221 (2012). 22699610 10.1038/nature11209 PMC3377744

[b62] Alekseyenko A. *et al.* Community differentiation of the cutaneous microbiota in psoriasis. Microbiome 1, 31 (2013). 24451201 10.1186/2049-2618-1-31 PMC4177411

[b63] Caporaso J. *et al.* QIIME allows analysis of high-throughput community sequencing data. Nat. Methods 7, 335–336 (2010). 20383131 10.1038/nmeth.f.303 PMC3156573

[b64] Edgar R. C. Search and clustering orders of magnitude faster than BLAST. Bioinformatics 26, 2460–2461 (2010). 20709691 10.1093/bioinformatics/btq461

[b65] Wang Q., Garrity G., Tiedje J. & Cole J. Naive Bayesian classifier for rapid assignment of rRNA sequences into the new bacterial taxonomy. Appl. Environ. Microbiol. 73, 5261–5267 (2007). 17586664 10.1128/AEM.00062-07 PMC1950982

[b66] Price M. N., Dehal P. S. & Arkin A. P. FastTree 2—approximately maximum-likelihood trees for large alignments. PLoS One 5, e9490 (2010). 20224823 10.1371/journal.pone.0009490 PMC2835736

[b67] Lozupone C. & Knight R. UniFrac: a new phylogenetic method for comparing microbial communities. Appl. Environ. Microbiol. 71, 8228–8235 (2005). 16332807 10.1128/AEM.71.12.8228-8235.2005 PMC1317376

[b68] Reynolds A., Richards G., de la Iglesia B. & Rayward-Smith V. Clustering rules: a comparison of partitioning and hierarchical clustering algorithms. J. Math. Model. Algorithm 5, 475–504 (1992).

[b69] Calinski R. & Harabasz J. A dendrite method for cluster analysis. Comm. Stat. 3, 1–27 (1974).

[b70] Dray S. & Dufour A.-B. The ade4 package: implementing the duality diagram for ecologists. J. Stat. Softw. 22, 1–20 (2007).

