# Supplementary material for: Metabolic and metagenomic outcomes from early-life pulsed antibiotic treatment
Source: Nat Commun. 2015 Jun 30;6:7486. doi: 10.1038/ncomms8486 (PMC4491183; doi:10.1038/ncomms8486)
Supplement: Supplementary Information — Supplementary Figures 1-10 and Supplementary Tables 1-2 [file ncomms8486-s1.pdf]

SUPPLEMENTARY FIGURES

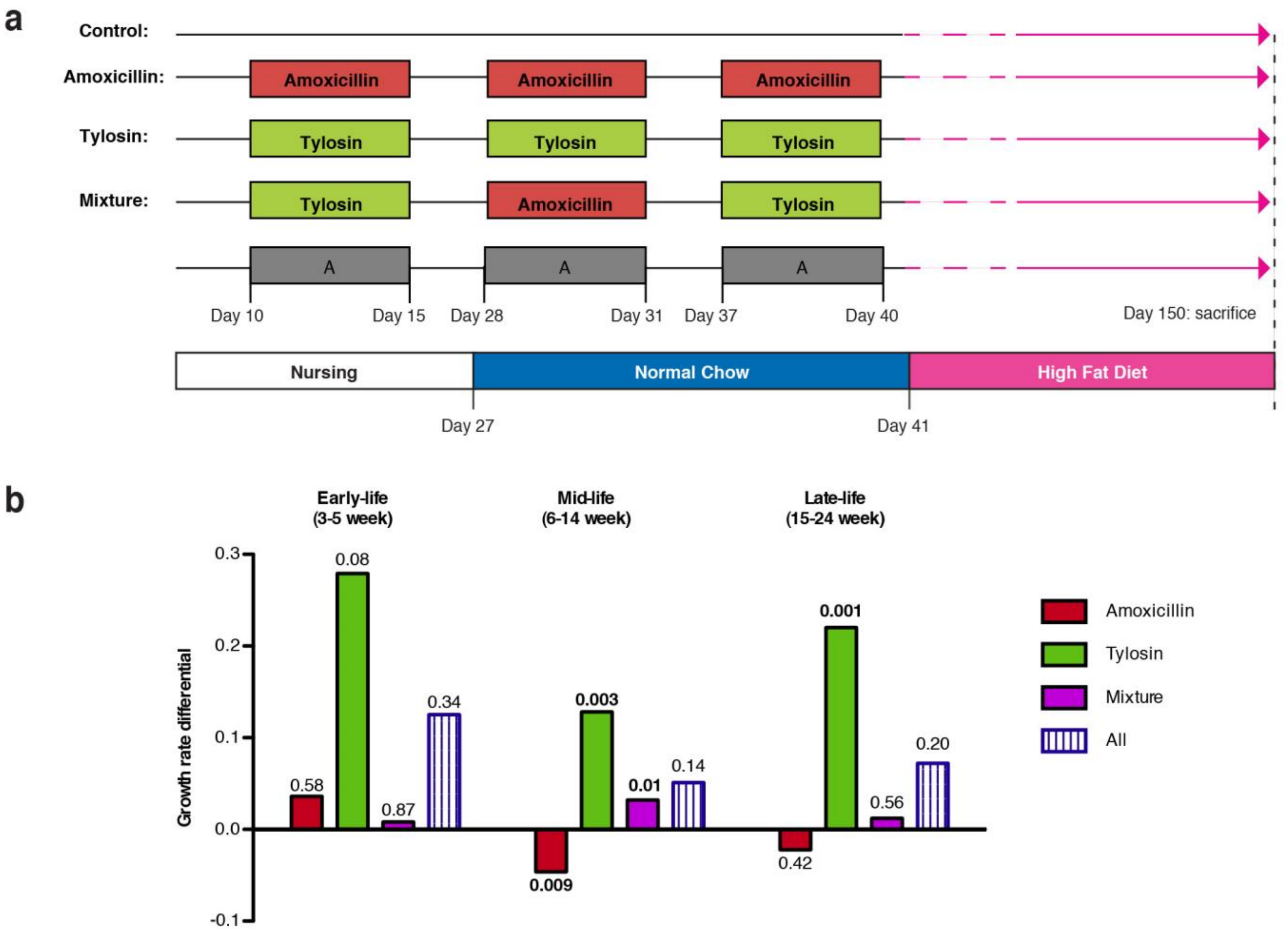

Supplementary Figure 1. Experimental design of Pulsed Antibiotic Treatment (PAT) study and effect on growth rate. a, In the PAT experiment, female C57/BL6J mice were bred and divided into control, amoxicillin, tylosin, and mixture study groups. Three antibiotic pulses (shown as A on second-to-bottom line) were provided via the drinking water to either the mothers if before weaning, or directly to the offspring if after weaning. All mice were switched from normal chow to high fat diet at day 41. Mice were sacrificed at approximately 150 days of life. Pink lines: high fat diet. **b,** Growth rate was determined by linear regression, and the growth rate differential of each antibiotic group compared to control was determined. Numbers represent p-values for significantly different growth rates (grams/day) from linear regression.

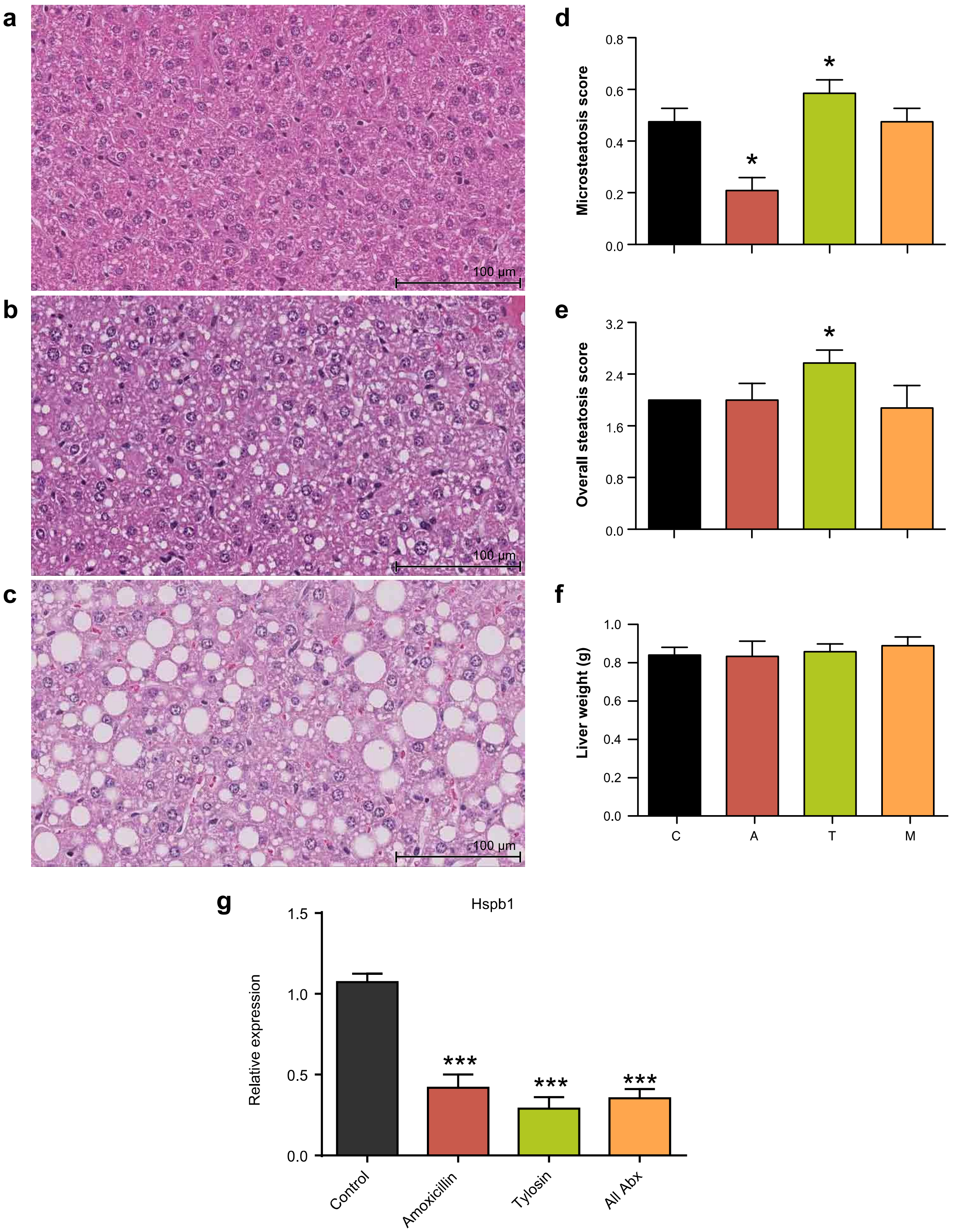

Supplementary Figure 2. The effect of PAT on liver histology and gene expression. a-c, Hepatic sections at 200x, stained with H&E, from mice sacrificed at 22 week of life (>16 weeks after last antibiotic exposure). a: No steatosis. b**:** Microsteatosis score = 1. c**:** Sample from one tylosin-treated mouse with notable macrosteatosis. **d,** Relative microsteatosis scores (Mean ± SD) across treatment groups. Scores evaluated by blind reading of 10 randomly selected high-power (200x) fields per mouse. Groups: C, control; A, amoxicillin; T, tylosin; M, mixture. **e,** Relative overall steatosis scores (Mean ± SD) were evaluated by reading each entire slide at 200x. **f,** Total liver weights (Mean ± SD) across treatment groups. * Indicates values significantly ($p<0.05$) different from control, Mann-Whitney U. **g,** Hepatic expression of Hspb1 at 22 weeks. Values in control mice, or mice receiving amoxicillin, tylosin, and either amoxicillin or tylosin (n= 3, 3, 3, and 6/group, respectively), measured by qPCR and normalized using GAPDH values. *** $p < 0.001$, one-way ANOVA with Dunnett's post hoc test.

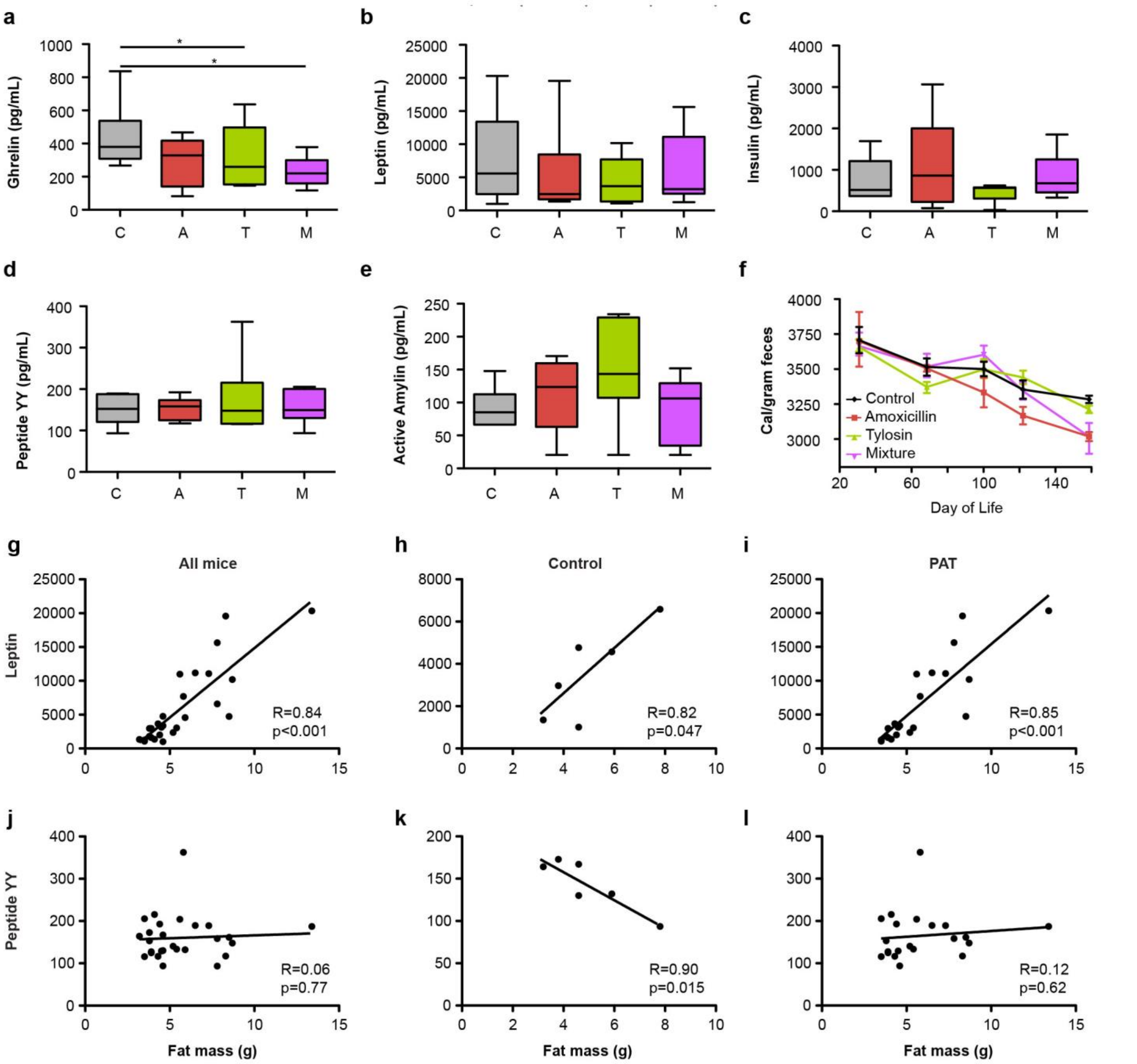

Supplementary Figure 3. Impact of PAT on serum metabolic hormone levels and calorie extraction. Serum hormone levels were assessed in samples collected at sacrifice, ~ day 150 of life. **a,** Ghrelin was significantly (*p<0.05, two-sided Wilcoxon Rank-Sum test) reduced in tylosin and mixture mice compared to controls. **b-e,** No significant differences were observed for serum leptin, insulin, peptide YY, or active amylin. **f,** Bomb calorimetry was used to measure calorie content at five time points in fecal pellets from control and PAT mice, bars represent SEM. Linear regression of fecal calories in each group over time demonstrated that fecal calories decreased over time, suggesting increased energy extraction with aging, with no significant differences between groups for control (R^2= 0.95, slope = -3.2±0.5, p = 0.006, significant for a slope of non-zero), amoxicillin (R^2= 0.99, slope = -5.4±0.3, p<0.001), tylosin (R^2= 0.67, slope = -2.7±1.1, p = 0.089), and mixture (R^2= 0.78, slope = -4.7±1.4, p = 0.046). **g-l,** Serum hormone concentration in relation to body fat at sacrifice. Serum digestive hormones leptin **(g-i)** and peptide YY **(j-l)** in all mice **(g, j)**, control mice only **(h, k)** and PAT-treated mice only **(i, l)**. Hormone levels were determined by Luminex Milliplex assay and correlated with fat content of mice at sacrifice, by linear regression analysis.

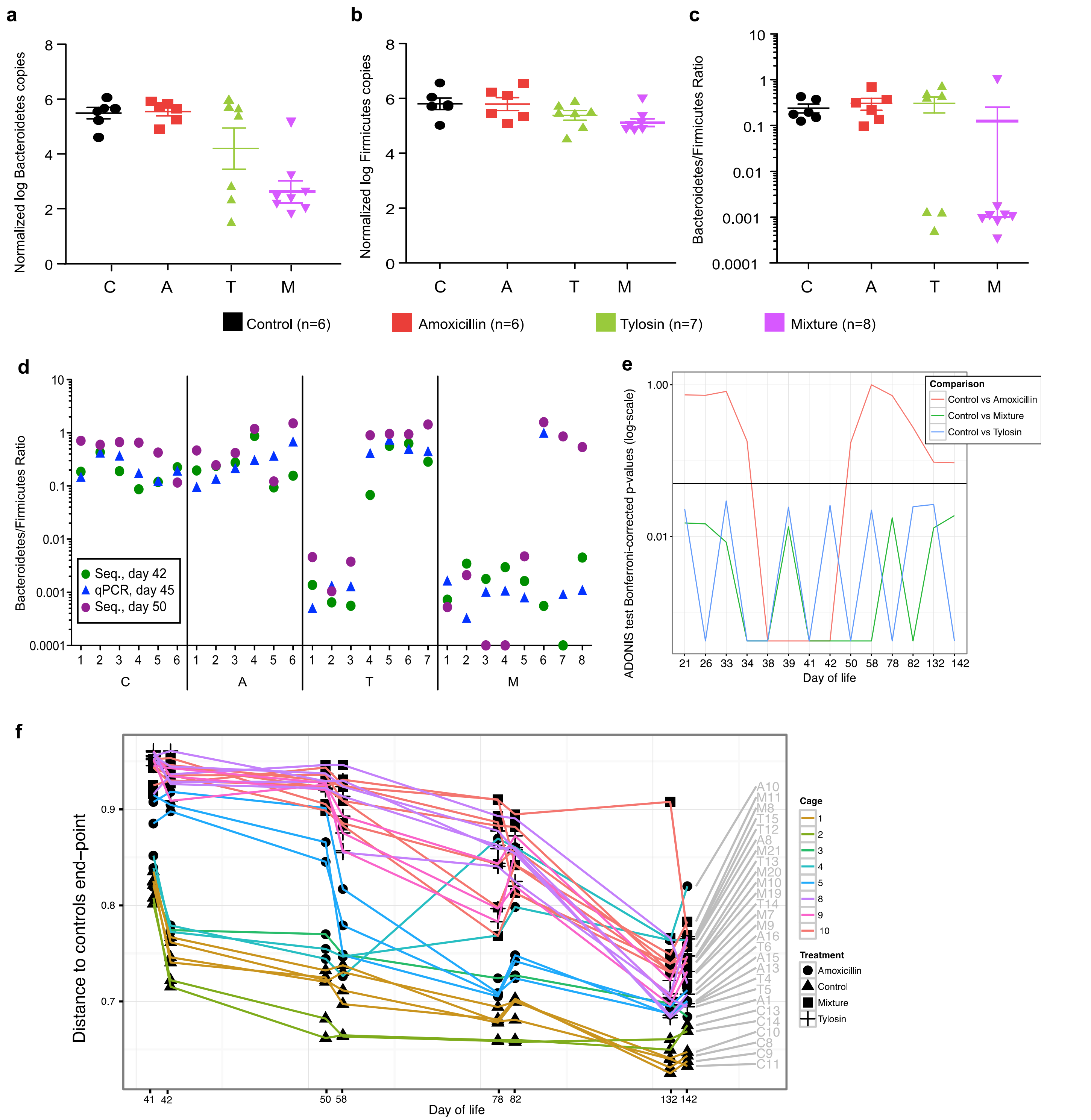

Supplementary Figure 4. Effect of PAT on intestinal microbiota composition. Gut microbial DNA samples from mothers, control mice, and PAT mice were collected from representative time points and examined by 454-pyrosequencing of the V1-V3 region of the 16S rRNA gene and by qPCR. **a-c,** Levels of Bacteroidetes, Firmicutes, and the Bacteroidetes/Firmicutes (B/F) ratio measured by qPCR at day 45 of life, four days after the final antibiotic pulse and three days after the initiation of the high fat diet. Values were normalized to DNA concentration (ng/μL). Horizontal lines represent mean values, and the bars indicate standard deviation. **d,** Validation of B/F ratio measurements by 454-pyrosequencing (Seq.) and qPCR in each mouse. qPCR was performed at day 45 of life, between 454-sequencing time points at days 42 and 50, following start of the high fat diet. Numbers refer to each of the experimental mice. Antibiotic treatment group: C (control); A (amoxicillin); T (tylosin); M (mixture). **e,** Bonferroni corrected ADONIS test-derived p-values for comparison of each of the antibiotic treatment groups to control group at all time points. **f,** Mean unweighted Unifrac distances between individual mice and the mice in the control group are plotted during the recovery phase (post-day 40) after antibiotic treatment has ceased. Each cage is indicated by color and each treatment group by symbol shape.

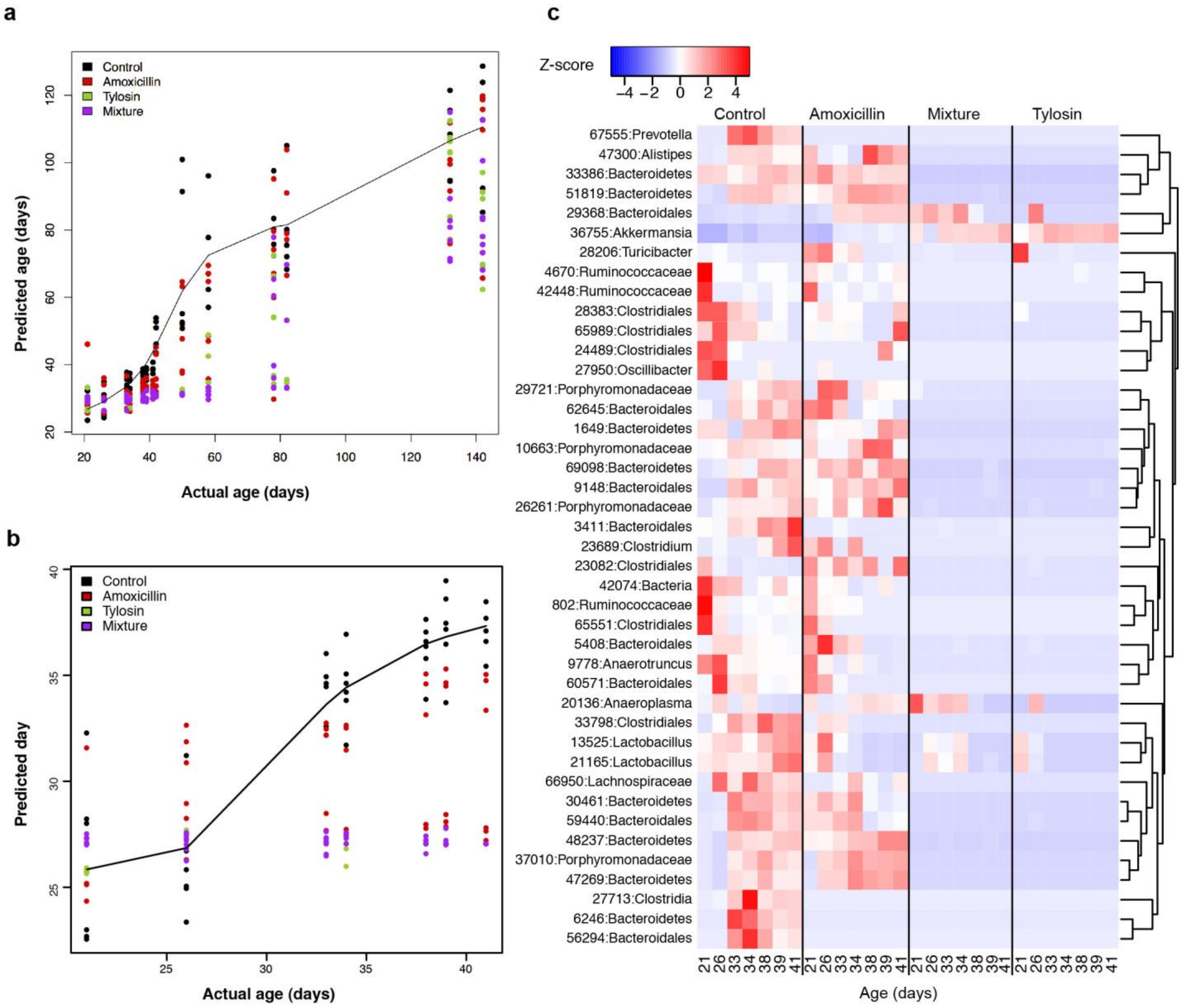

Supplementary Figure 5. The effect of PAT on microbiota maturity prediction. A random forest regression model was trained on the control microbiota over time to calculate the maturity index as a function of predicted chronological age. **a,** Independent estimation of day of life (predicted day of life) by the maturity model versus actual day of life of individual mice, when the model was trained on all time points. **b-c,** To determine which OTUs were important in early-life, before the dietary change, a second regression model was trained on the control samples for day of life 21 to 41. b: Predicted day of life based on the maturity model trained on the pre-HFD time points. c: The heat map displays abundance of OTUs important for determining maturity in the first 41 days of life on normal chow, according to group.

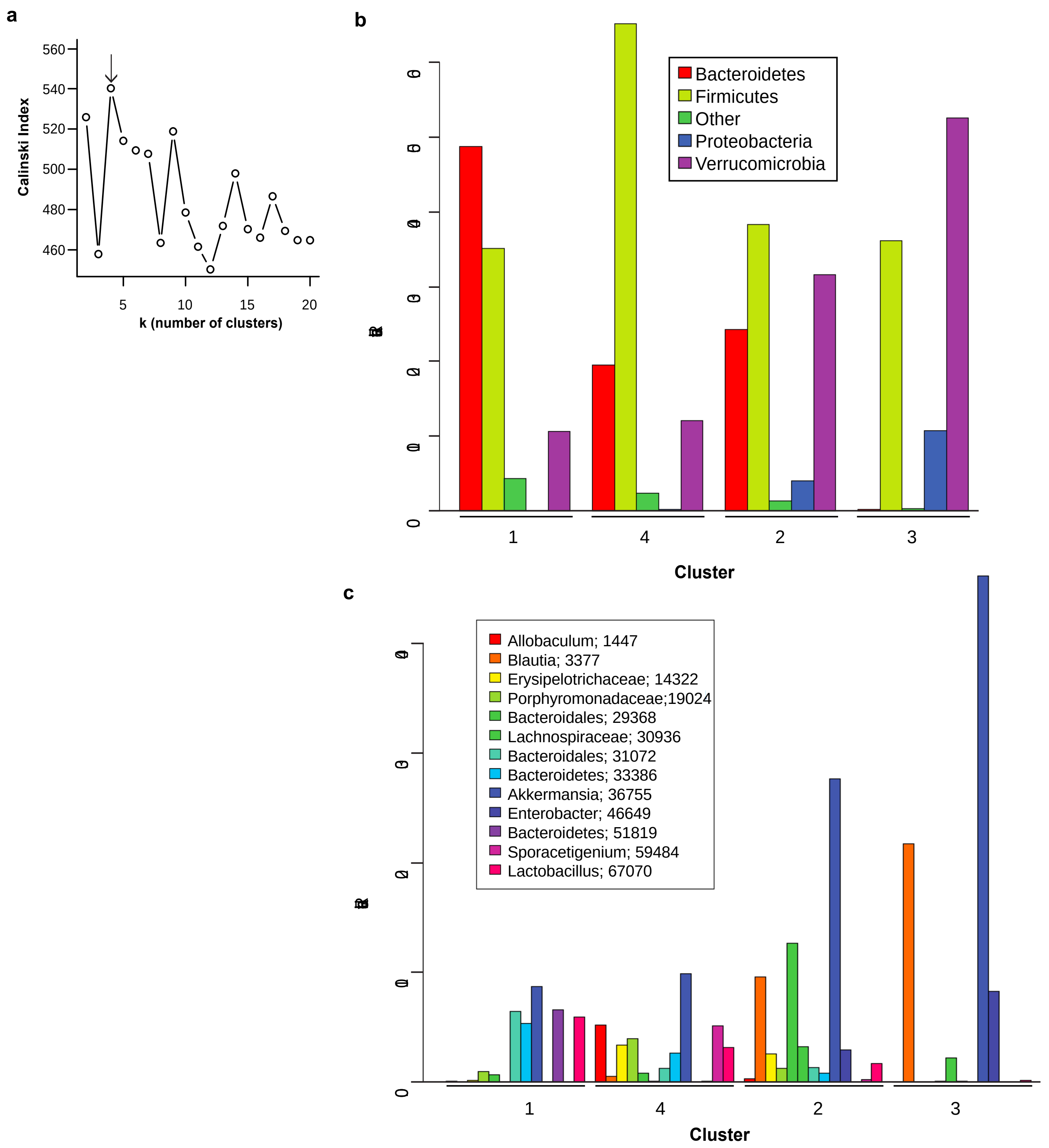

Supplementary Figure 6. Clustering analysis of 338 specimens over the course of the study. a, Calinski index of the strength of clustering, according to number of clusters. For the 338 fecal specimens studied by 454-pyrosequencing of the 16S rRNA gene from extracted DNA, the highest Calinski index was for four clusters. **b,** Phylum-level and **c,** OTU-level composition of the four distinct taxonomic clusters.

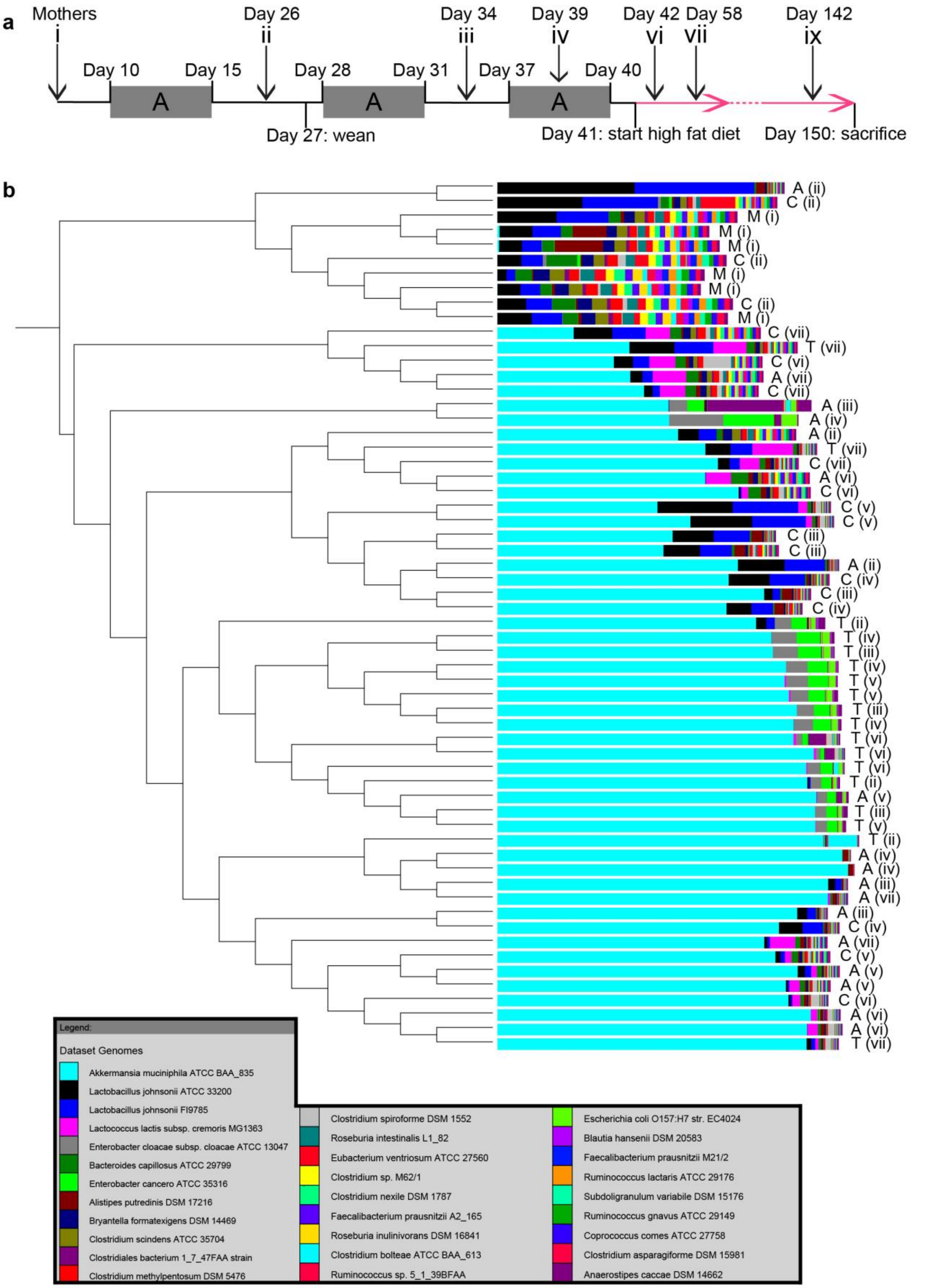

Supplementary Figure 7. Phylogenetic map of fecal samples from mothers, control, and PAT mice. a, Three representative control, tylosin, and amoxicillin mice at 6 time points each, and 6 samples from mothers underwent metagenomic analysis using 100 base pair reads generating 5 GB/sample, for a total of 300 GB**. b,** Phylogenetic map constructed from reads aligned to a Reference Genome Database. C=control, A=amoxicillin, T=tylosin, M=mother.

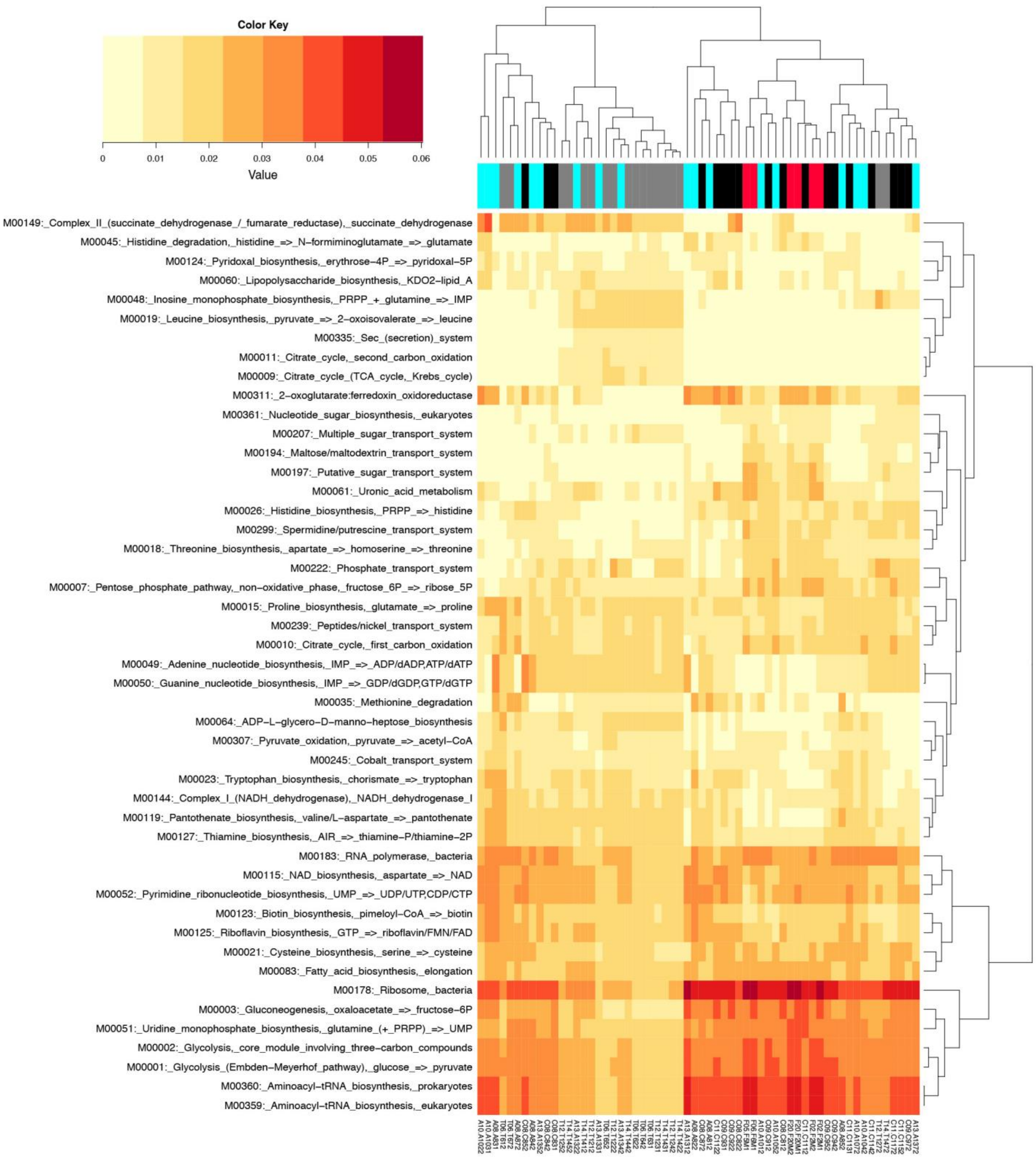

Supplementary Figure 8. Metagenomic KEGG module abundance, by unsupervised clustering. Modules analyzed were those in the 25th percentile that were highest in variance. Samples are color-coded by group: mothers, red; control, black; amoxicillin, blue; tylosin, gray.

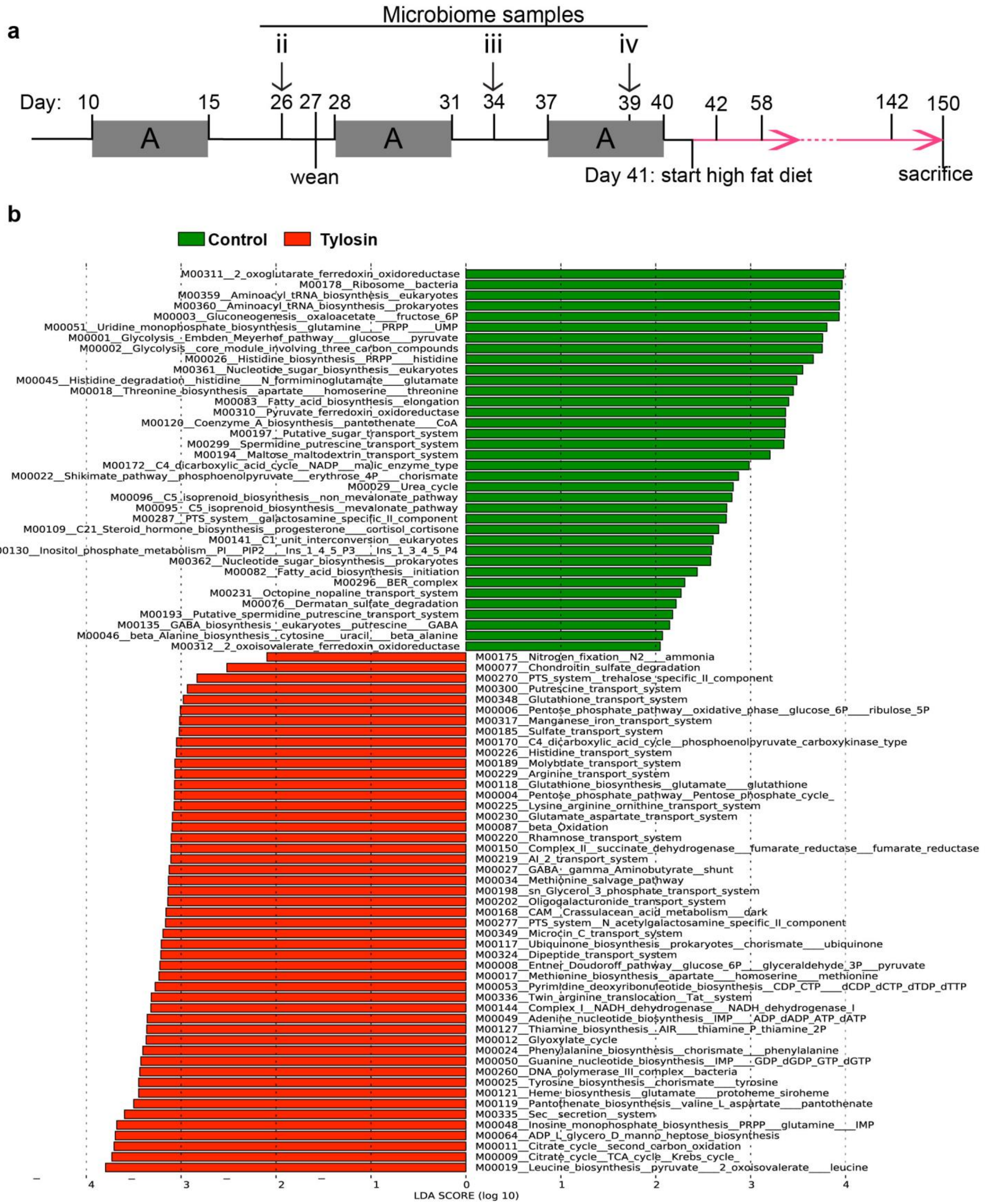

Supplementary Figure 9. KEGG modules differing in control and PAT mice in early life. a, Time points of fecal specimens sampled for early-life metagenomic analysis; all points are before the initiation of the high fat diet on day 41**. b,** Modules significantly ($p<0.05$) up-regulated in control (green) or tylosin (red) samples determined by LEfSe analysis. In contrast, comparison of control vs. amoxicillin yielded no modules with significantly different expression (data not shown).

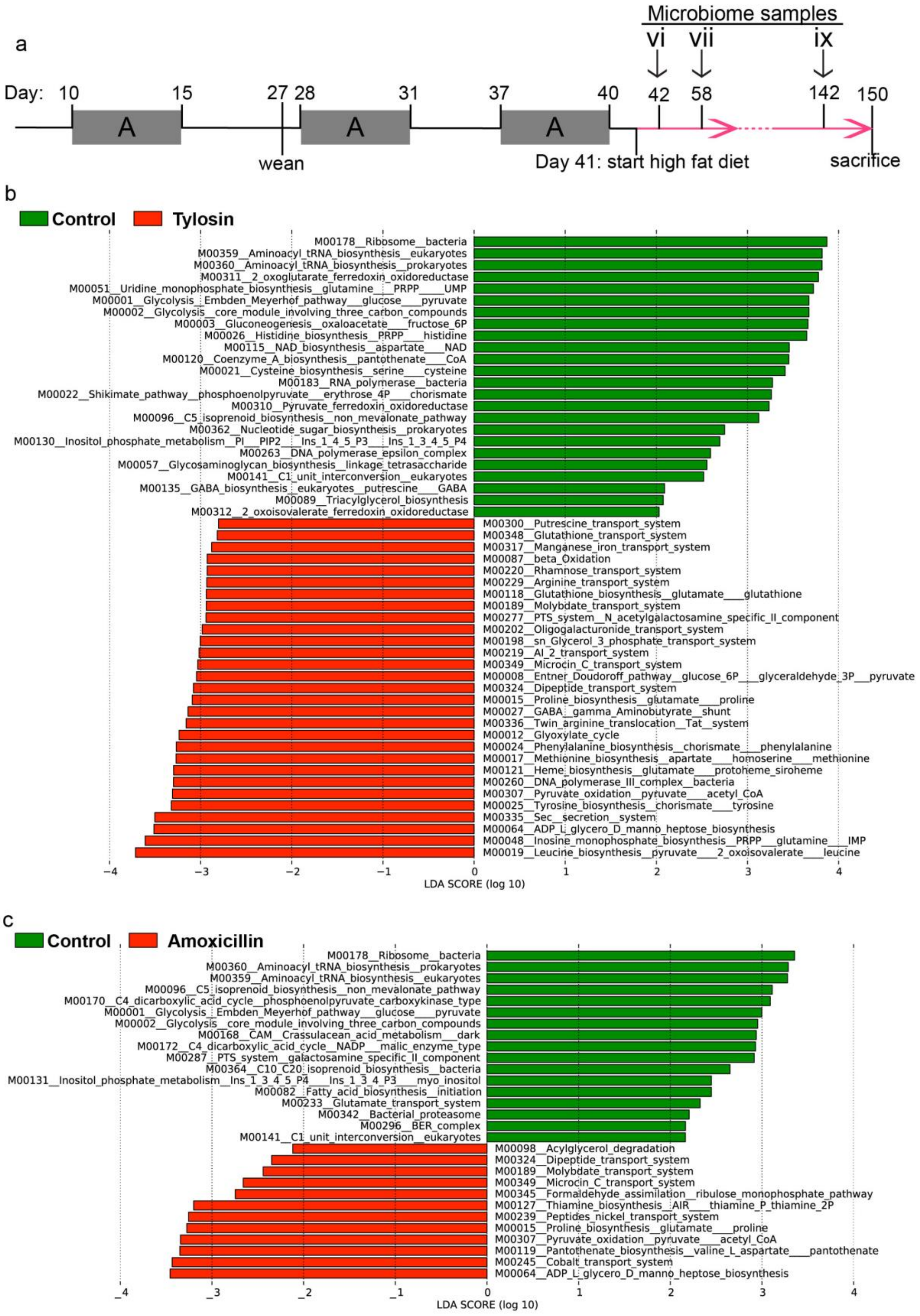

Supplementary Figure 10. KEGG modules differing in control and PAT mice later in life. a, Time points of fecal specimens sampled for late-life metagenomic analysis; all time points are after the initiation of the high fat diet on day 41. **b,** Modules significantly ($p<0.05$) up-regulated in control (green) or tylosin (red) samples determined by LEfSe analysis. **c,** Modules significantly ($p<0.05$) up-regulated in control (green) or amoxicillin (red) samples determined by LEfSe analysis.

SUPPLEMENTARY TABLES

Supplementary Table 1. KEGG modules significantly[a] and consistently altered by PAT

Category	Sub-category	Module	Name	TE[b]	TL	AL
Carbohydrate and lipid metabolism	Central carbohydrate metabolism	M00001	Glycolysis (Embden-Meyerhof pathway), glucose => pyruvate	Down	Down	Down
		M00002	Glycolysis, core module involving three-carbon compounds	Down	Down	Down
		M00003	Gluconeogenesis, oxaloacetate => fructose-6P	Down	Down	
		M00310	Pyruvate:ferredoxin oxidoreductase	Down	Down	
		M00311	2-oxoglutarate:ferredoxin oxidoreductase	Down	Down	
		M00312	2-oxoisovalerate:ferredoxin oxidoreductase	Down	Down	
		M00008	Entner-Doudoroff pathway, glucose-6P => glyceraldehyde-3P + pyruvate	Up	Up	
		M00307	Pyruvate oxidation, pyruvate => acetyl-CoA		Up	Up
	Other carbohydrate metabolism	M00012	Glyoxylate cycle	Up	Up	
	Lipopolysaccharide synthesis	M00064	ADP-L-glycero-D-manno-heptose biosynthesis	Up	Up	Up
	Fatty acid metabolism	M00087	beta-Oxidation	Up	Up	
		M00082	Fatty acid biosynthesis, initiation	Down		Down
	Lipid metabolism	M00130	Inositol phosphate metabolism, PI=> PIP2 => Ins	Down	Down	
	Terpenoid backbone biosynthesis	M00096	C5 isoprenoid biosynthesis, non-mevalonate pathway	Down	Down	Down
Energy metabolism	Carbon fixation	M00172	C4-dicarboxylic acid cycle, NADP - malic enzyme type	Down		Down
		M00168	CAM (Crassulacean acid metabolism), dark	Up		Down
		M00170	C4-dicarboxylic acid cycle, phosphoenolpyruvate carboxykinase type	Up		Down
Metabolism	Aminoacyl tRNA	M00359	Aminoacyl-tRNA biosynthesis, eukaryotes	Down	Down	Down
		M00360	Aminoacyl-tRNA biosynthesis, prokaryotes	Down	Down	Down
	Nucleotide sugar	M00362	Nucleotide sugar biosynthesis, prokaryotes	Down	Down	
Nucleotide and amino acid metabolism	Histidine metabolism	M00026	Histidine biosynthesis, PRPP => histidine	Down	Down	
	Aromatic amino acid metabolism	M00022	Shikimate pathway, phosphoenolpyruvate + erythrose-4P => chorismate	Down	Down	
		M00024	Phenylalanine biosynthesis, chorismate => phenylalanine	Up	Up	
		M00025	Tyrosine biosynthesis, chorismate => tyrosine	Up	Up	
	Branched-chain amino acid metabolism	M00019	Valine/isoleucine biosynthesis, pyruvate => valine / 2-oxobutanoate => isoleucine	Up	Up	
	Arginine and proline metabolism	M00015	Proline biosynthesis, glutamate => proline		Up	Up
	Cysteine and methionine metab.	M00017	Methionine biosynthesis, apartate => homoserine => methionine	Up	Up	
	Other amino acid metabolism	M00027	GABA (gamma-Aminobutyrate) shunt	Up	Up	
	Polyamine biosynthesis	M00135	GABA biosynthesis, eukaryotes, putrescine => GABA	Down	Down	
	Cofactor and vitamin biosynthesis	M00120	Coenzyme A biosynthesis, pantothenate => CoA	Down	Down	
		M00141	C1-unit interconversion, eukaryotes	Down	Down	Down
		M00118	Glutathione biosynthesis, glutamate => glutathione	Up	Up	
		M00121	Heme biosynthesis, glutamate => protoheme/siroheme	Up	Up	
		M00119	Pantothenate biosynthesis, valine/L-aspartate => pantothenate	Up		Up
		M00127	Thiamine biosynthesis, AIR => thiamine-P/thiamine-2P	Up		Up
	Purine metabolism	M00048	Inosine monophosphate biosynthesis, PRPP + glutamine => IMP	Up	Up	
	Pyrimidine metabolism	M00051	Uridine monophosphate biosynthesis, glutamine (+ PRPP) => UMP	Down	Down	
Environmental Information processing	Phosphotransferase system (PTS)	M00287	PTS system, galactosamine-specific II component	Down		Down
		M00277	PTS system, N-acetylgalactosamine-specific II component	Up	Up	
	Bacterial secretion system	M00335	Sec (secretion) system	Up	Up	
		M00336	Twin-arginine translocation (Tat) system	Up	Up	
	Metallic cation and iron-siderophore system	M00317	Manganese/iron transport system	Up	Up	
	Mineral and organic ion transport system	M00189	Molybdate transport system	Up	Up	Up
		M00300	Putrescine transport system	Up	Up	
	Peptide and nickel transport system	M00324	Dipeptide transport system	Up	Up	Up
		M00349	Microcin C transport system	Up	Up	Up
		M00348	Glutathione transport system	Up	Up	
	Amino acid transport system	M00229	Arginine transport system	Up	Up	
	Saccharide, polyol, and lipid transport system	M00198	Putative sn-glycerol-phosphate transport system	Up	Up	
		M00202	Oligogalacturonide transport system	Up	Up	
		M00219	AI-2 transport system	Up	Up	
		M00220	Rhamnose transport system	Up	Up	
Genetic information processing	DNA polymerase	M00260	DNA polymerase III complex, bacteria	Up	Up	
	Repair system	M00296	BER complex	Down		Down
	Ribosome	M00178	Ribosome, bacteria	Down	Down	Down

[a] KEGG Modules significantly different in ≥two tests by LEfSe analysis, control (early in life) vs. tylosin (early in life), control (late in life) vs. tylosin (late in life), or control (late in life) vs. amoxicillin (late in life). Up or down refers to increased or decreased levels in the antibiotic treatment group with reference to the control, respectively.

[b] TE = Tylosin early time points, corresponds to Figure S9.
TL = Tylosin late time points, corresponds to Figure S10b.
AL = Amoxicillin late time points, corresponds to Figure S10c.

Supplementary Table 2. Illumina sequencing metrics.

Sample Name	Input reads	Mouse-free reads	Number clean reads in fasta	Number reads with KEGG hits	% of reads with KEGG hits
PAAD-A10-A1012	39,186,492	38,907,124	36,826,571	8,826,723	23.97
PAAD-A10-A1022	42,817,920	41,704,446	38,919,378	9,745,006	25.04
PAAD-A10-A1031	50,313,006	49,821,748	45,777,016	12,546,584	27.41
PAAD-A10-A1042	68,407,298	61,094,800	55,495,106	16,694,492	30.08
PAAD-A10-A1052	58,681,658	51,407,580	47,063,700	11,642,395	24.74
PAAD-A10-A1072	55,438,780	42,829,856	37,022,958	10,665,274	28.81
PAAD-A13-A1312	47,587,118	47,205,222	39,660,826	10,425,572	26.29
PAAD-A13-A1322	48,507,696	48,487,616	41,505,699	12,416,656	29.92
PAAD-A13-A1331	48,832,028	48,758,544	45,044,412	19,453,110	43.19
PAAD-A13-A1342	50,698,872	50,326,552	46,278,025	19,125,349	41.33
PAAD-A13-A1352	63,245,272	56,206,550	50,961,453	15,261,912	29.95
PAAD-A13-A1372	65,834,486	56,213,008	50,429,775	12,180,362	24.15
PAAD-A8-A812	37,113,612	36,047,128	33,881,598	9,694,634	28.61
PAAD-A8-A822	44,203,356	43,622,116	40,732,276	10,859,642	26.66
PAAD-A8-A831	48,753,334	48,615,788	44,628,584	12,781,310	28.64
PAAD-A8-A842	58,331,676	53,176,820	48,634,326	14,011,830	28.81
PAAD-A8-A852	55,136,218	29,399,158	26,774,318	8,015,798	29.94
PAAD-A8-A872	61,650,168	44,096,464	39,183,365	11,581,258	29.56
PAAD-C11-C1112	38,245,326	34,486,858	32,541,395	7,554,677	23.22
PAAD-C11-C1122	45,923,466	42,995,648	40,343,840	10,915,558	27.06
PAAD-C11-C1131	42,550,292	41,695,310	38,712,914	11,286,589	29.15
PAAD-C11-C1142	59,025,730	56,006,472	51,462,223	15,590,871	30.3
PAAD-C11-C1152	56,914,656	36,852,584	34,286,066	8,896,188	25.95
PAAD-C11-C1172	53,828,078	47,654,418	43,323,499	10,511,469	24.26
PAAD-C8-C812	34,687,810	32,849,174	31,050,702	8,139,679	26.21
PAAD-C8-C822	43,848,430	43,251,864	40,526,087	11,031,537	27.22
PAAD-C8-C831	47,994,146	44,517,576	41,644,795	11,266,087	27.05
PAAD-C8-C842	57,821,420	49,114,408	45,166,039	13,568,972	30.04
PAAD-C8-C852	56,713,180	46,204,354	42,763,322	12,132,735	28.37
PAAD-C8-C872	59,028,358	53,126,206	48,583,813	12,974,332	26.71
PAAD-C9-C912	38,421,762	25,650,288	24,189,191	5,807,586	24.01
PAAD-C9-C922	40,707,674	30,228,476	28,428,407	7,122,863	25.06
PAAD-C9-C931	51,075,926	48,033,100	44,764,125	12,066,997	26.96
PAAD-C9-C942	62,754,326	55,493,252	51,125,699	14,160,533	27.7
PAAD-C9-C952	58,479,004	51,098,154	47,237,102	12,474,900	26.41
PAAD-C9-C972	59,928,962	51,753,948	46,677,713	11,675,089	25.01
PAAD-F2-F2M1	50,318,970	47,954,368	43,929,768	10,604,470	24.14
PAAD-F2-F2M2	34,229,962	32,565,396	30,789,699	7,646,826	24.84
PAAD-F20-F20M1	49,797,074	47,048,562	42,847,538	10,267,532	23.96
PAAD-F20-F20M2	36,492,080	34,996,568	32,957,150	8,136,363	24.69
PAAD-F5-F5M1	50,318,416	48,340,218	44,728,249	10,526,455	23.53
PAAD-F6-F6M1	46,309,506	38,697,218	34,377,657	7,439,013	21.64
PAAD-T12-T1212	37,478,440	36,759,812	34,476,227	11,662,676	33.83
PAAD-T12-T1222	44,710,574	42,302,616	39,068,636	17,336,557	44.37
PAAD-T12-T1231	56,082,892	55,108,538	49,937,184	23,911,628	47.88
PAAD-T12-T1242	54,044,208	36,646,474	33,766,533	15,058,366	44.6
PAAD-T12-T1252	61,695,976	50,058,962	45,686,281	15,949,229	34.91
PAAD-T12-T1272	62,097,420	51,537,674	46,218,202	13,232,043	28.63
PAAD-T14-T1412	38,123,502	35,598,602	33,276,657	10,587,815	31.82
PAAD-T14-T1422	47,714,508	47,001,144	43,153,902	19,412,219	44.98
PAAD-T14-T1431	55,541,730	54,929,928	49,703,811	23,149,530	46.57
PAAD-T14-T1442	56,841,400	52,175,122	47,996,505	19,352,416	40.32
PAAD-T14-T1452	60,276,758	53,575,886	48,851,489	16,745,647	34.28
PAAD-T14-T1472	64,031,502	46,789,512	42,273,616	10,697,134	25.3
PAAD-T6-T612	53,258,976	53,159,110	45,305,104	16,913,076	37.33
PAAD-T6-T622	45,824,840	43,200,664	40,027,024	17,859,284	44.62
PAAD-T6-T631	46,700,710	46,257,070	42,769,463	19,520,243	45.64
PAAD-T6-T642	52,621,376	49,017,928	44,728,374	21,187,858	47.37
PAAD-T6-T652	62,110,078	57,346,380	51,243,812	22,767,523	44.43
PAAD-T6-T672	63,861,774	59,979,260	53,419,226	14,934,945	27.96
**Average/sample**	**51,386,170**	**45,999,660**	**42,052,974**	**13,133,390**	**30.92**
